# Isolation of a Novel Flavanonol and an Alkylresorcinol with Highly Potent Anti-Trypanosomal Activity from Libyan Propolis

**DOI:** 10.3390/molecules24061041

**Published:** 2019-03-15

**Authors:** Weam Siheri, Godwin U. Ebiloma, John O. Igoli, Alexander I. Gray, Marco Biddau, Pilaslak Akrachalanont, Samya Alenezi, Mohammad A. Alwashih, RuAngelie Edrada-Ebel, Sylke Muller, Catherine E. Lawrence, James Fearnley, David G. Watson, Harry P. De Koning

**Affiliations:** 1 Strathclyde Institute of Pharmacy and Biomedical Science, University of Strathclyde, 161 Cathedral Street, Glasgow G4 0RE, UK; weamsiheri@gmail.com (W.S.); igolij@gmail.com (J.O.I.); a.i.gray@strath.ac.uk (A.I.G.); pilaslak.a@dmsc.mail.go.th (P.A.); s-alenezi@strath.ac.uk (S.A.); ruangelie.edrada-ebel@strath.ac.uk (R.E.-E.); catherine.lawrence@strath.ac.uk (C.E.L.); 2 Institute of Infection, Immunity and Inflammation, College of Medical, Veterinary and Life Sciences, University of Glasgow, Glasgow G12 8TA, UK; godwin4godwin@gmail.com (G.U.E.); mark.biddau@gmail.com (M.B.); sylkemuller@hotmail.co.uk (S.M.); 3 Department of Chemistry, University of Agriculture, Makurdi PMB 2373, Nigeria; 4 General Directorate of Medical Services, Ministry of Interior, Riyadh 13321, Saudi Arabia; mweshyyeh@yahoo.com; 5 BeeVital, Whitby, North Yorkshire YO22 5JR, UK; james.fearnley@beearc.com

**Keywords:** propolis, *Trypanosoma brucei*, anthelminthic activity, metabolomics, alkylresorcinol, bilobol

## Abstract

Twelve propolis samples from different parts of Libya were investigated for their phytochemical constituents. Ethanol extracts of the samples and some purified compounds were tested against *Trypanosoma brucei*, *Plasmodium falciparum* and against two helminth species, *Trichinella spiralis* and *Caenorhabditis elegans*, showing various degrees of activity. Fourteen compounds were isolated from the propolis samples, including a novel compound Taxifolin-3-acetyl-4′-methyl ether (**4**), a flavanonol derivative. The crude extracts showed moderate activity against *T. spiralis* and *C. elegans*, while the purified compounds had low activity against *P. falciparum*. Anti-trypanosomal activity (EC_50_ = 0.7 µg/mL) was exhibited by a fraction containing a cardol identified as bilobol (**10**) and this fraction had no effect on Human Foreskin Fibroblasts (HFF), even at 2.0 mg/mL, thus demonstrating excellent selectivity. A metabolomics study was used to explore the mechanism of action of the fraction and it revealed significant disturbances in trypanosomal phospholipid metabolism, especially the formation of choline phospholipids. We conclude that a potent and highly selective new trypanocide may be present in the fraction.

## 1. Introduction

Several studies have confirmed the activity of propolis and its components against protozoan parasites, particularly kinetoplastids such as *Trypanosoma brucei*, *Leishmania donovani* and *Trypanosoma cruzi*, which cause sleeping sickness, visceral leishmaniasis and Chagas disease respectively [1,2,3,4]. Recent studies have also observed antiprotozoal effects of propolis extracts against *Plasmodium falciparum*, *Plasmodium malariae*, *Plasmodium vivax* and *Plasmodium ovale*, the parasite species that cause malaria [5,6]. Samples of propolis have also been found to be effective against *Entamoeba histolytica* and *Guardia lamblia*, which cause intestinal infections [7,8], and against multicellular organisms such as intestinal worms including, cestodes (tapeworms), nematodes (roundworms) and trematodes (e.g., *Schistosoma* spp.) [9,10]. In the present study we have carried out further characterization and tests on Libyan propolis. Results of previous studies on Libyan propolis are summarized in Table 1 and included reports of biological activities varying from antioxidant to hypolipidaemic and hepatoprotective. We have previously reported the isolation of four compounds isolated from Libyan propolis and performed preliminary tests for their anti-trypanosomal and anti-leishmanial activity [4,6]. The current manuscript reports a much more detailed characterization of the propolis extracts and identifies 14 additional purified compounds from the Libyan propolis, several of which have not been reported before in the literature. These were tested for activity against the helminths *Trichinella spiralis* and *Caenorhabditis elegans*, against *P. falciparum* and against drug-sensitive and resistant strains of *Trypanosoma brucei*.

## 2. Results

### 2.1. Anti-Helminthic Activity of the Propolis Samples

Crude extracts of the propolis samples were tested against helminths (Table 2). Samples P1–P5 showed promising activity against *T. spiralis* but samples P6–P12 all gave <20% inhibition. This anthelminthic activity appeared to be species-specific as none of the samples displayed only weak inhibition of *C. elegans* (<20%) at 1 µg/mL and 10 µg/mL.

### 2.2. Isolation and Characterization of Compounds from Samples P1, P2, P7 and P9

**Figure 1:**
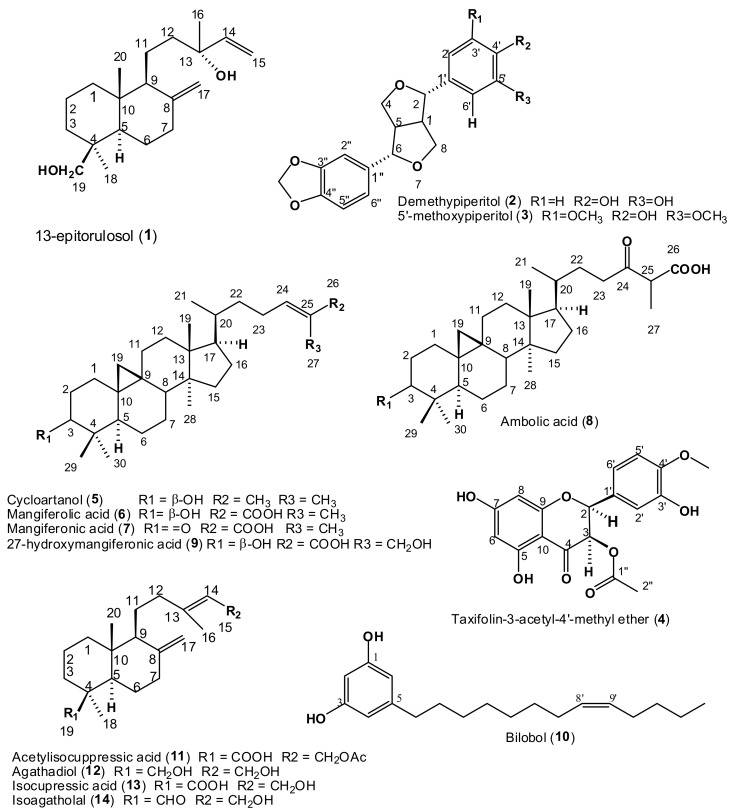
Structures of the compounds isolated from Libyan propolis in this study.

The isolation and characterization of three diterpenes and a lignan from sample P1 was reported in our previous paper [4]. In the current study 14 additional compounds (Table 3) were isolated and characterized by comparison of their ^1^H- and ^13^C-NMR spectra to literature data (Tables S1–S14); their structures are given in Figure 1.

### 2.3. Characterization of Compound ***4*** as 3-Acetoxy-5,7,3′-trihydroxy-4′-methoxyflavanone or Taxifolin-3-acetyl-4′-methyl ether

The NMR data and the optical rotation value [α] = +1.6 for this compound suggest the same configuration as reported for (2*R*,3*R*)-taxifolin-3-acetate isolated from *Chrysothamnus viscidiflorus* ssp. viscidiflorus by Stevens et al. [15]. These authors also isolated a methyl ether that was identified as taxifolin-3-acetate-x′-methyl ether since they could not establish the position of the methoxy moiety at -C-3′ or C-4′. In the current study the structure of compound **4** (Figure 1) was determined by NMR, COSY and mass spectrometry (Figures S1–S5). ^1^H-NMR data for **4** showed a highly deshielded and H-bonded proton at δ 11.52 (s), a methoxy at δ 3.85 and an acetoxy at δ 1.96. It also showed two coupled protons on oxygen-bearing carbons at δ 5.20 (d, *J* = 11.7 Hz) and δ 5.75 (d, *J* = 11.7 Hz), four sets of aromatic protons made up of two meta-split protons at δ 6.10 and 6.05, typical of the A-ring protons of a flavonoid, and two multiplets, at δ 6.93 (1H, m) and δ 6.86 (2H, m) (Table S1). The HSQC spectrum (Figure S4), shows the attachment of the carbon-bonded protons to their respective carbons. The HMBC spectrum (Figure S5), revealed that the de-shielded phenolic proton (δH 11.52) coupled to three aromatic carbons, 97.4 (C-6), 101.8 (C-10) and 164.4 (C-5). The protons at δ 6.10 (1H, d, *J* =2.4 Hz) (H-6) and 6.05 (H-8) (1H, d, *J* = 2.3 Hz) showed 3*J* correlations to C-10 and to each other’s carbons and weak correlations 2*J* to a carbon at 164.4 ppm (C-7) while H-8 had a weak correlation to 163.1 ppm (C-9). The proton doublet at δ 5.75(1H, d, *J* = 11.7 Hz) (attached to C-3 at 72.2 ppm) was coupled (COSY) to another proton doublet at δ 5.20 (attached to C-2 at 81.5 ppm), and they had HMBC correlations to a carbonyl at 169.2 ppm which also had coupling from the methyl at 1.96 ppm, indicative of the 3-acetoxy moiety. The proton at δ 5.20 coupled to C-3 and C-4 (191.7 ppm) and to 109.3, 121.9, and 127.0 ppm, C-6′, C-2′ and C-1′, respectively. The protons attached to carbons C-6’ and C-2’ at δ 6.93 and δ 6.86 coupled with the carbon at 81.5 ppm (C-2) and to each other’s carbon (109.3 and 121.9 ppm) and 146.7 ppm (C-4’) which also had coupling from the 4’-OCH3 at δ 3.85 ppm. All assignments were made as given in Table S1. Also evident in the spectrum is a fatty contamination with signals between δ 0.50 and 2.60 ppm. This was a mixture of common fatty acids that are not known to have any antimicrobial activity; we were unable to further purify **4** from this fatty residue as all such attempts resulted in the loss of the compound together with the contaminants.

### 2.4. Testing of the Antiparasite Activity of the Isolated Compounds

Ten of the isolated compounds were tested against pentamidine-resistant and -sensitive strains of *T. brucei.* The results for the activity against *T. brucei* (s427) and pentamidine resistant strain *T. brucei* B48 showed that the isolated compounds exhibited significant activity against *Trypanosoma* spp., with the lowest EC_50_, 0.70 µg/mL, being observed for cardol **10** (Table 4). B48 cells were equally sensitive to all the propolis-derived compounds, but displayed >1000-fold resistance to pentamidine and were also resistant to melaminophenyl arsenical drugs [16].

Some of the isolated compounds were tested also against *P. falciparum*. The highest activity was again obtained for the cardol fraction. The activity of the isolated compounds was much lower against *P. falciparum* than against *T. brucei*. The anthelminthic activity of some of the compounds isolated from propolis was tested against *T. spiralis*. The results indicate that only compounds **13** and **14** had low levels of anti-helminthic activity (31.8 ± 3.4 and 36.1 ± 5.6% inhibition, respectively, at 1 µg/mL). Compounds **1** and **4**–**8** had no activity against *T. spiralis*, nor did the two labdane diterpenes, 13-epirutosolal and 13-epicupressic acid, isolated from P1 in our previous study [4].

### 2.5. Metabolomic Profiling of the Effect of the Cardol-Rich Fraction 10 against T. brucei

**Figure 2:**
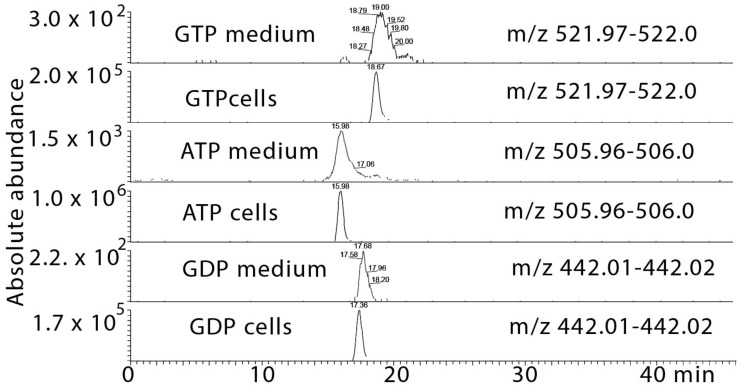
Extracted ion traces showing leakage of small amounts of high energy phosphates GTP, ATP and GDP into the growth medium in the treated cells.

Metabolomics testing of the cardol-rich fraction was carried out with trypanosomes with the assumption this would reveal cardol-specific effects as the mangiferonic acid contaminant had low activity on its own. Three control samples were compared with four samples treated with the cardol fraction; the positive ion mode data is shown in Table S15. With this number of samples, it is difficult to be certain that data are normally distributed and that *p*-values are valid. However, one might hope to see changes of several metabolites within the same class or biochemical pathway, highlighting a clear theme in the metabolite changes. Indeed, we found that many long chain lipids are depleted, and there are changes in several phosphocholine and phosphoserine lipids containing highly unsaturated fatty acids with chain lengths ≥ C_20_. Corresponding elevations in choline and CDP-choline suggest that the cells might be countering the effects of the treatment by increasing these intermediates. The negative ion data shown in Table S16 revealed that the abundance of several phosphatidyl glycerol (PG) and phosphatidyl inositol (PI) lipids was lowered by the treatment. Thus, the treatment seems to be depleting the cells of a wide range of lipids. Comparison of the growth medium for treated versus control indicates that many of the lipids which are reduced in the cell extracts were greatly elevated in the growth medium. This is more marked for the negative ion data where there are many PI and PG lipids which are at much higher levels in the medium from the treated trypanosomes (Table S17). However, there are also some of lipids observed in positive mode in the cells that are lowered and that are correspondingly elevated in the growth medium (Table S18). In addition, there are a number of fatty acids that are elevated to some extent in the growth medium of the treated versus the control cells (Table S17). The presence of lipids in the growth medium may be due to cell lysis though the viability of the cells post-treatment did not indicate extensive cell death. There were traces of high energy phosphates (GTP, GDP, ATP, Figure 2, Table S17) in the growth medium of the treated cells and again this could be due to cell lysis. However, the trypanosomes contain oxidised trypanothione as typical intra-cellular product and there is no evidence of this being released into the growth medium.

## 3. Discussion

One of the most consistent of the many biological activities of propolis, regardless of its quite different compositions and origins, is its antiprotozoal activity. Clearly, bees target plants that can afford them this type of biological protection. Anthelminthic testing of propolis has been much less extensively studied, although there are some previous studies that have observed this activity [10]. In this study the in vitro anthelminthic activity of Libyan propolis against *T. spiralis* and *C. elegans* was assessed. Samples P1–P4, at a concentration of 10 µg/mL, exhibited 61–80% inhibition against *T. spiralis*. We previously reported that these propolis samples have low toxicity against mammalian cells [6] and they might thus have utility to purge *T. spiralis* from the intestine. The crude extracts of P6-P12 were not active against *T. spiralis* but compounds **13** and **14** isolated from sample P9 had some weak and variable activity against *T. spiralis* and the results suggest that propolis affords less protection against helminths than against protozoa, which are the more dangerous pathogens for bees.

Samples P1 and P2 had a composition typical of Mediterranean propolis [17], which has been reported to exhibit biological properties such as antioxidant, anti-proliferative, anti-inflammatory and had neuroprotective effects. The botanical origin of such propolis samples has yet to be identified, but the diterpenic profile suggests that the source plant should be in the Cupressaceae family, which is common in the region. Diterpenes such as 13-epitorulosol (**1**) and 13-epicupressic acid were also isolated from the leaves of Cupressaceae family member *Cryptomeria japonica* [18].

MPLC fractionation of P7, from the tropical region of Southeast Libya, was carried out and this resulted in the isolation of a number cycloartane triterpenes: cycloartanol (**5**), mangiferolic acid (**6**), mangiferonic acid (**7**), ambolic acid (**8**) and 27-hydroxymangeferonic acid (**9**); these were previously isolated from Cameroonian propolis [19]. Most of the compounds isolated from it displayed significant anti-protozoal activity, especially **5**. Many studies on propolis from different African regions such as Kenya, Cameroon, Congo and Ethiopia, showed that triterpenoids are major chemical components [20,21]. Three more cycloartane-type triterpenes were isolated and identified from East Java propolis, and *Macaranga tanarius* L. parasol tree and *Mangifera indica* L. (mango) were named as the plant sources [22]. In addition, a fraction containing about 60% cardol (**10**) was isolated from P7. This compound was isolated previously from hydrogenated cashew nut shell [23] and from Brazilian propolis [24]. Of all the isolated compounds tested, this compound has the highest anti-trypanosomal activity, despite the fact that the crude propolis sample had relatively low activity. Importantly, the compounds isolated from the propolis samples reported in this work had no measurable effects on the HFF human cell line at up to 2 mg/mL, demonstrating excellent selectivity.

Metabolomic profiling of the effects of the cardol rich fraction suggested that it might be targeting the cell membrane and acting rather like a surfactant, extracting lipid from the membrane, making it leaky. Thus, its mode of action might be similar to that of Miltefosine an established anti-leishmanial drug that changes microbial membrane fluidity [25]. Alkyl resorcinols have been found to promote leakage of electrolytes from erythrocytes [26] and this is consistent with the leakage of high energy phosphates observed in the current data. Alkyl resorcinols have been found to be potent inhibitors of phospholipase Cγ1 which is responsible for hydrolysis of phosphoinositol lipids releasing diacylglycerol and phosphoinositol [27], but the current data do not indicate such an effect in *T. brucei* since the treatment lowered several PI lipids. Alkyl resorcinols have been found to have antibacterial, antifungal and anti-helminthic activity while having low toxicity towards mammals [26].

In conclusion, Libyan Propolis shows activity against a range of parasites. Its production is commercially viable and it is non-destructive since bees collect it without damaging the plants they collect from. The main problem of exploiting the biological activity of a natural product such as propolis is to guarantee a standardized supply of material. In addition, some of the active compounds isolated from propolis could be used as lead compounds for producing more potent drugs. The exact purpose and usage of this intriguing material still have to be fully elucidated. One thing is clear propolis is always biologically active and can potentially be harvested in large amounts in an environmentally friendly way. Moreover, the most active compound, cardol, also known as bilobol, is easily synthesized and derivatized, and thus open to optimization [28].

## 4. Materials and Methods

### 4.1. Reagents and Materials

Absolute ethanol, HPLC grade acetonitrile, hexane, methanol, formic acid and syringe filters (Acrodisc^®^) were obtained from Fisher Scientific (Loughborough, UK). Chloroform, dimethyl sulfoxide (DMSO), deuterated chloroform, DMSO-*d*_6_, silica gel 60 (0.04–0.06 mm mesh size) and Wilmad NMR tubes were obtained from Sigma-Aldrich (Gillingham, Dorset, UK). HPLC grade water was produced in-house using a Milli Q system. Twelve propolis samples were collected from different localities in Libya. Six silica gel cartridges (24 g) for the Revelris MPLC system were obtained from Alltech Ltd. (Carnforth, Lancs, UK). The origin of the propolis samples was described in detail in a previous paper [6].

### 4.2. Extraction of Propolis Samples

Using sonication at 50 °C for 1 h, 20 g of each propolis sample (P1–P12) was extracted thrice with 100 mL ethanol. The extracts were filtered and combined after removal of solvents with a rotary evaporator. The solvent-free extracts were re-dissolved in 5 mL ethyl acetate and stored for further use.

### 4.3. Column Chromatography

Crude P1 extract (2.0 g) was fractionated on silica gel (50 g) using varying proportions of hexane/ethyl acetate in a stepwise gradient. Fractions of 50 mL were collected and the second fraction P1-2 (252 mg) and third fraction P1-3, (111 mg), both eluted with hexane/ethyl acetate (90:10), were selected for further purification using medium-pressure liquid chromatography (MPLC). The ethanolic extract of P2 was similarly fractionated and fraction P2-12 (354 mg, eluted with hexane/ethyl acetate (60:40)) and fraction P2–24 (204 mg; hexane/ethyl acetate (30:70)) were also selected for further purification.

### 4.4. Purification of Column Fractions Using MPLC

Separation by MPLC was carried out on a Grace Reveleris system (Alltech Ltd.) fitted with commercially packed silica or C_18_ silica cartridges (Alltech Ltd.). P1-3 (1 g) was dissolved in hexane: EtOAc (50:50), absorbed on 2 g of Celite, and introduced into a dry loader for the MPLC. A 24.0 g silica column and 12 mL/min flow rate was used; program: 100% hexane to hexane: ethyl acetate (80:20) in 30 min, then to 100% ethyl acetate at 50 min, yielding 17.7 mg of 13-epitorulosol (**1**) from P1. Column fraction P1-2 was loaded onto the MPLC system with a 12 g silica gel cartridge and eluted with a stepwise gradient of ethyl acetate in hexane, yielding 17.8 mg of taxifolin-3-acetate-4′-methylether (**4**). The same procedure yielded 22.7 mg of demethylpiperitol (**2**) from 354 mg of P2-12 (354 mg). Fraction P2-24 (208 mg) was eluted from a Grace 12 g C-18 cartridge with acetonitrile: water (40:60, 12 mL/min, 30 min; linear increase to 100% acetonitrile over 30 min), yielding 20.3 mg of 5′-methoxypiperitol (**3**). Fractionation of the ethanol extract of sample P7 (1 g) on the MPLC system fitted with a 24 g silica gel cartridge (100% hexane to 100% ethyl acetate over 60 min; flow rate of 24 mL/min), yielded six compounds: 25.7 mg of cycloartanol (**5**), 37.8 mg of a fraction containing cardol (**10**) and mangiferolic acid (**6**), 29.2 mg of mangiferolic acid (**6**), 21.7 mg of mangiferonic acid (**7**), 41.8 mg of ambolic acid (**8**), 33.8 mg of 27-hydroxymangiferonic acid (**9**). Fractionation of the ethanol extract of P9 (1 g) yielded four compounds. 27.1 mg acetylisocupressic acid (**11**), 25.4 mg of agathadiol (**12**), 22.3 mg of isocupressic acid (**13**) and 22.2 mg of isoagatholal (**14**).

### 4.5. Liquid Chromatography-High Resolution Mass Spectrometry (LC-HRMS) and HPLC with UV/Evaporative Light Scattering Detection (ELSD)

Profiling of the ethanolic extract of propolis (EEP) and selected sub-fractions from MPLC was carried out using an Agilent 1100 HPLC system (Agilent Technologies LDA UK Ltd, Stockport, Cheshire, UK) linked to a Shodex ELSD (SEDEX75, SEDERE, Olivet, France) and an Agilent UV detector set at 290 nm. The sample extracts were dissolved in water-acetonitrile (70:30) at a concentration of 0.5 mg/mL. An ACE-C18 column (150 × 3 mm, 3 µm) (HiChrom, Reading, UK) was employed for separation with mobile phases A (water) and B (ACN) at a flow rate of 300 µL/min. The gradient elution was 0–15 min gradient, 30–50% B; 15–25 min, 50% B; 25–40 min gradient, 50–70% B; 40–50 min, 70% B; 50–51 min increasing to 100% B; 51–59 min 100% B; 60–70 min back to 30% B. Propolis fractions obtained from column chromatography and MPLC were dissolved in methanol (1 mg/mL) and 10 μL of the solutions were subjected to LC-MS (LTQ Orbitrap (Thermo Fisher, Perth, Scotland, UK) in negative ion mode with a needle voltage of −4.0 kV, and sheath and auxiliary gas flows of 50 and 10 arbitrary units, respectively. Separation was carried out using an ACE-C18 column (150 × 3 mm, 3 µm); mobile phase: 0.1% *v*/*v* formic acid in water (A) and 0.1% *v*/*v* formic acid in ACN (B), flow rate 300 µL/min. The same gradient program as above was used.

### 4.6. Nuclear Magnetic Resonance (NMR) Spectroscopy

Samples were dissolved in DMSO-*d*_6_ or CDCl_3_ and ^1^H-, ^13^C- and 2D-NMR spectra (COSY, HMBC, HSQC) were acquired on an AV600 or AV400 FTNMR spectrophotometer (Bruker, Coventry, UK).

### 4.7. Software and Data Processing for LC-HR-MS

Xcalibur 2.2 from Thermo Fisher Scientific was used to check the raw LC-HRMS and GC-MS data and generate the MS-based chromatograms shown in the manuscript. Clarity from DataApex (Prague, Czech Republic) was used to handle the LC-UV-ELSD data.

### 4.8. In Vitro Testing against T. brucei and P. falciparum and Cytotoxicity

Cytotoxicity [29] and testing against *T. brucei* and *P. falciparum* [6] was carried out as described previously.

### 4.9. In Vitro Anti-Helminthic Activity of P1–P12 Against Trichinella spiralis

Female and male C57BL/6 mice aged 8–12 weeks and 20–25 g in weight, were kept under conventional animal house conditions in accordance with the Home Office regulations in the animal unit at the University of Strathclyde and the terms of the Home Office license (Number P894DB549). Experimental groups consisted of a minimum of five mice. Infective muscle larvae were obtained from digestion of infected mice (>30 days post infection). Mice were killed by CO_2_ inhalation and skins, snouts, extremities and abdominal organs were removed. The carcasses were cut into pieces and homogenized in a Kenwood blender. The material was digested in 200 ml 0.9% NaCl/0.5% Pepsin/0.5% HCl solution/mouse at 37 °C under agitation for 1.5 h, and filtered through a coarse sieve (mesh 1 mm) to remove undigested tissue and bone. The larvae were collected by a series of three successive washings and sedimentations in 0.9% NaCl, suspended in 50 mL 0.9% NaCl, and counted using a Leica light microscope. Experimental mice were infected orally with 400 larvae in 200 μL 0.1% agarose culture medium.

The resazurin assay was performed in 96-well culture plates. *T. spiralis* was prepared as described above. 1% *v*/*v* of 10,000 units/mL penicillin and 10 mg/mL streptomycin (Sigma-Aldrich, Poole, UK) were added to eliminate any bacterial contamination. Prior to the experiment, propolis samples were diluted to 1 and 10 µg/mL in culture medium. 100 µL of worms and 100 µL of propolis samples, medium or known anthelminthic (nitazoxanide (3.13 μg/mL) or levamisole (2.1 μg/mL); both Sigma-Aldrich) were added to each well of a 96-well culture plate. Resazurin (Cambridge Bioscience, Cambridge, UK; 125 mg/L) was added aseptically at 10% *v*/*v* culture medium at various time points. The plate was incubated at 37 °C in 5% CO_2_ at various time points. Resazurin reduction was monitored spectrophotometrically at 570 nm and 600 nm over a period of time with absorbance taken at specific time intervals over the period, blanking with medium only. Resazurin reduction by viable parasites was expressed as % reduction as a function of incubation time, and quantified by fluorescence at λ_ex_ = 544 nm; λ_em_ = 590–610 nm (Polarstar, BMG Labtech, Aylesbury, UK). The effect of drug treatment on parasite survival was determined as the mean suppression of fluorescence compared to control values.

### 4.10. Metabolomics Experiments

*T. brucei* bloodstream forms of strain T. brucei 427 were grown in 75 cm^2^ flasks with HMI-9 medium, supplemented with fetal calf serum (FCS, 10%, *v*/*v*), incubated at 37 °C and 5% CO_2_ for two days until mid-log phase. Cultures were then treated with 0.5 × EC50 of the test samples and incubated for 10 h (37 °C, 5% CO_2_). Parasite viability was verified at the end of the incubation and was not significantly different between control and treated cultures. From each culture 1 × 10^8^ cells/sample were transferred to a 50 mL falcon tube and quenched by rapidly cooling cells to 4 °C on dry ice/ethanol before centrifugation (1250× *g*, 4 °C, 10 min). The cells were re-suspended in 1 mL of phosphate buffered saline (PBS) at 4 °C, transferred to an Eppendorf tube, pelleted (1250× *g*, 4 °C, 10 min), washed twice in 1 mL of PBS at 4 °C and finally resuspended in 200 µL of chloroform: methanol: water (1:3:1 (CMW)) at 4 °C, mixing well. These samples were left on a see-saw rocking shaker in the cold room (4 °C) for 1 h at maximum speed. In addition, 10 µL of the spent medium from each treatment was mixed with 200 µL CMW and centrifuged (13,000 rpm, 10 min, 4 °C); 180 µL of the supernatant was flushed with argon before storage at −80 °C. LC-MS analysis was carried out using a ZIC-pHILIC column (150 × 4.6 mm, 5 µm) connected to a mass spectrometer as described previously [30]. The data was extracted by using *m*/*z* Mine 2.14 [31]. Most metabolites were identified to MSI level 2 (exact mass matching to <3 ppm) with some being annotated at MSI level 1 where retention time was matched to that of an authentic standard.

## Figures and Tables

**Table 1:** Summary of previous studies on Libyan propolis.

Propolis Origin (Number of Samples)	Extract Investigated	Analysis Conducted	Biological Activity Observed
Surman City, West Libya, (One sample)	Ethanol extract/CAPE	TLC investigation	Cytotoxicity and Antioxidant activity [11]
Zawia City, West-Libya, (One sample)	Ethanol extract	Partial purification	Inhibitory effect against *S. aureus* [12]
Alaquria and Tokra, North East Libya (Two samples)	Ethanol extract and purified compounds	LC-MS-GC-MS, HPLC-UV/ELSD, NMR, diterpenes, lignin compounds	High activity against *T. brucei*, *L. donovani* [4]
Surman City, West Libya, (One sample)	Aqueous extract	None	*In vivo* hypolipidaemic and antiatherogenic effects in mice [13]
Surman City, West Libya (One sample)	Aqueous extract	None	Hepatoprotective and hypolipidemic effects in guinea pigs [14]
Different Geographic areas West, East, South East and South West of Libya (12 samples)	Ethanol extract	LC-MS, PCA, analysis combined with HCA	Activity against *T. brucei*, *L. donovani*, *P. falciparum*, *C. fasciculata* and *M. marinum* [6]

CAPE = Caffeic acid phenethyl ester.

**Table 2:** Anthelminthic activity of crude propolis extracts P1–P5 against *T. spiralis* (*n* = 3).

Propolis Samples	Inhibition of *T. spiralis* 1 µg/mL	10 µg/mL
P1	19.5 ± 3.5	63.1 ± 0.5
P2	38.9 ± 0.1	57.2 ± 6.4
P3	51.9 ± 0.1	63.6 ± 0.3
P4	59.3 ± 0.1	61.3 ± 0.7
P5	4.77 ± 0.21	56.2 ± 5.2
Nitazoxanide ^a^	28.7 ± 11.8	
Levamisole ^b^	56.7 ± 3.9	

^a^ 3.13 µg/mL; ^b^ 2.1 µg/mL.

**Table 3:** Isolated compounds from samples P1, P2, P7 and P9.

No.	Yield (mg)	Name	Propolis Sample	Molecular Formula	*m*/*z* -Ve Ion	Class of Compound
(**1**)	17.7	13-Epitorulosolol	P1	C_20_H_34_O_2_	305.4812	Diterpene
(**2**)	22.7	Demethylpiperitol	P2	C_19_H_18_O_6_	341.1211	Lignan
(**3**)	20.3	5′-Methoxypiperitol	P2	C_21_H_22_O_7_	385.1136	Lignan
(**4**)	17.7	Taxifolin-3-acetyl-4′methyl ether	P1	C_18_H_16_O_8_	359.0766	Flavanone
(**5**)	25.7	Cycloartanol	P7	C_30_H_50_O	425.3821	Cycloartane triterpene
(**6**)	29.2	Mangiferolic acid	P7	C_30_H_48_O_3_	455.7123	Cycloartane triterpene
(**7**)	21.7	Mangiferonic acid	P7	C_30_H_46_O_3_	453.6934	Cycloartane triterpene
(**8**)	41.8	Ambolic acid C_31_H_51_O_3_	P7	C_31_H_50_O_3_	469.729	Cycloartane triterpene
(**9**)	33.8	27-Hydroxymangiferonic acid	P7	C_30_H_46_O_4_	469.6982	Cycloartane triterpene
(**10**)	37.8	Cardol plus mangiferolic acid (6)	P7	C_21_H_34_O_2_	317.2489, 455.7123	Resorcinol, Cycloartane
(**11**)	27.1	Acetylisocupressic acid	P9	C_20_H_32_O_3_	319.1711	Diterpene
(**12**)	25.4	Agathadiol	P9	C_20_H_34_O_2_	305.4838	Diterpene
(**13**)	22.3	Isocupressic acid	P9	C_20_H_32_O_3_	319.4791	Diterpene
(**14**)	22.2	Isoagatholal	P9	C_20_H_32_O_2_	303.2412	Diterpene

**Table 4:** EC_50_ values (µg/mL) of the antiprotozoal activities of compounds isolated from Libyan propolis tested against *T. brucei* and *P. falciparum*.

Compound	*T. brucei* (s427) ^a^	*T. brucei* (B48) ^a^	*P. falciparum* ^a^
(**2**)	2.7 ± 0.2	2.68 ± 0.04	17.5 ± 0.1
(**3**)	13.1 ± 0.1	12.4 ± 1.6	-
(**5**)	3.7 ± 0.1	3.42 ± 0.08	*
(**6**)	-	-	*
(**7**)	14.6 ± 0.2	14.7 ± 0.4	49.2 ± 9.5
(**8**)	**-**	**-**	*
(**9**)	35.2 ± 0.6	34.9 ± 0.3	*
(**10**)	0.70 ± 0.03	0.70 ± 0.06	12.4 ± 2.1
(**11**)	25.0 ± 0.2	25.6 ± 0.9	-
(**12**)	7.0 ± 0.6	6.90 ± 0.45	-
(**13**)	3.0 ± 0.1	2.73 ± 0.11	*
(**14**)	10.4 ± 0.1	10.2 ± 0.8	
Pentamidine ^1^	0.00012 ± 0.00003	0.255 ± 0.009	-
Chloroquine ^1^	-	-	0.0034 ± 0.00003

* Not active at 0.1 mg/ml. ^a^ Average of EC_50_ (µg/mL) ± Standard error of mean (*n* = 3) for the isolated compounds; **-** not tested. ^1^ for the control compounds pentamidine and chloroquine the EC_50_ was expressed as µM.

## Supplementary Materials

The supplementary materials are available online.

## References

[1] Almutairi S., Eapen B., Chundi S.M., Akhalil A., Siheri W., Clements C., Fearnley J., Watson D.G., Edrada-Ebel R. (2014). New anti-trypanosomal active prenylated compounds from African propolis. Phytochem. Lett..

[2] Falcão S.I., Vale N., Cos P., Gomes P., Freire C., Maes L., Vilas-Boas M. (2014). In vitro evaluation of Portuguese propolis and floral sources for antiprotozoal, antibacterial and antifungal activity. Phytother. Res..

[3] Omar R.M., Igoli J., Gray A.I., Ebiloma G.U., Clements C., Fearnley J., Ebel R.A., Zhang T., De Koning H.P., Watson D.G. (2016). Chemical characterisation of Nigerian red propolis and its biological activity against *Trypanosoma brucei*. Phytochem. Anal..

[4] Siheri W., Igoli J.O., Gray A.I., Nasciemento T.G., Zhang T., Fearnley J., Clements C.J., Carter K.C., Carruthers J., Edrada-Ebel R. (2014). The isolation of antiprotozoal compounds from Libyan propolis. Phytother. Res..

[5] Olayemi K.I. (2014). Therapeutic potentials of Nigerian insect-propolis against the malarial parasite, *Plasmodium berghei* (Haemosporida: Plasmodidae). Am. J. Drug Disc. Dev..

[6] Siheri W., Zhang T., Ebiloma G.U., Biddau M., Woods N., Hussain M.Y., Clements C.J., Fearnley J., Ebel R.E., Paget T. (2016). Chemical and antimicrobial profiling of propolis from different regions within Libya. PLoS ONE.

[7] Abdel-Fattah N.S., Nada O.H. (2007). Effect of propolis versus metronidazole and their combined use in treatment of acute experimental giardiasis. J. Egypt Soc. Parasitol..

[8] Alday-Provencio S., Diaz G., Rascon L., Quintero J., Alday E., Robles-Zepeda R., Garibay-Escobar A., Astiazaran H., Hernandez J., Velazquez C. (2015). Sonoran propolis and some of its chemical constituents inhibit in vitro growth of *Giardia lamblia* trophozoites. Planta Med..

[9] Hassan S.E., Abou-El-Dobal S.K., Hegazi A.G. (2016). Bioassay of Egyptian propolis on *Toxocara vitulorum* adult worms. World Appl. Sci. J..

[10] Siheri W., Alenezi S., Tusiimire J., Watson D.G. (2017). The chemical and biological properties of propolis. Bee Products-Chemical and Biological Properties.

[11] Abd El-Rahman S.S. (2010). West-Libyan propolis and rosemary have synergistic anti-tumor effect against 12-O-tetradecanoylphorbol 13-acetate-induced skin tumor in BULB/C mice previously initiated with 7,12-dimethylbenz [a] anthracene. Basic Appl. Pathol..

[12] Sarkez N.H. (2014). Antimicrobial properties of Libyan propolis against *Staphylococcus aureus*. Libyan J. Med. Res..

[13] Azab E.A., Algridi M.A., Lashkham N.M. (2015). Hypolipidemic and antiatherogenic effects of aqueous extract of Libyan propolis in lead acetate intoxicated male albino mice. IJSR.

[14] Azab A.E., Lashkham N.M., Albasha M.O. (2015). Haematoprotective and hypolipidemic effects of aqueous extract of Libyan propolis against sodium nitrite induced haematotoxicity and hyperlipidemia in Guinea pigs. Am. J. Biosci. Bioeng..

[15] Stevens J.F., Wollenweber E., Ivancic M., Hsu V.L., Sundberg S., Deinzer M.L. (1999). Leaf surface flavonoids of *Chrysothamnus*. Phytochemistry.

[16] Bridges D., Gould M.K., Nerima B., Mäser P., Burchmore R.J.S., De Koning H.P. (2007). Loss of the high affinity pentamidine transporter is responsible for high levels of cross-resistance between arsenical and diamidine drugs in African trypanosomes. Mol. Pharmacol..

[17] Popova M.P., Graikou K., Chinou I., Bankova V.S. (2010). GC-MS profiling of diterpene compounds in Mediterranean propolis from Greece. J. Agric. Food Chem..

[18] Su W., Fang J., Cheng Y. (1994). Labdanes from *Cryptomeria japonica*. Phytochemistry.

[19] Kardar M.N., Zhang T., Coxon G.D., Watson D.G., Fearnley J., Seidel V. (2014). Characterisation of triterpenes and new phenolic lipids in Cameroonian propolis. Phytochemistry.

[20] Petrova A., Popova M., Kuzmanova C., Tsvetkova I., Naydenski H., Muli E., Bankova V. (2010). New biologically active compounds from Kenyan propolis. Fitoterapia.

[21] Zhang T., Omar R., Siheri W., Al Mutairi S., Clements C., Fearnley J., Edrada-Ebel R., Watson D. (2014). Chromatographic analysis with different detectors in the chemical characterisation and dereplication of African propolis. Talanta.

[22] Trusheva B., Popova M., Koendhori E.B., Tsvetkova I., Naydenski C., Bankova V. (2011). Indonesian propolis: Chemical composition, biological activity and botanical origin. Nat. Prod. Res..

[23] Maia F.J.N., Ribeiro V.G.P., Almeida M.O., Lomonaco D., Mafezoli J., Mazzetto S.E. (2013). Study of antioxidant activity of a phenyl phosphorylated compound derived from hydrogenated cardol by thermogravimetric analysis. Br. J. Appl. Sci. Technol..

[24] Silva M.S.S., De Lima S.G., Oliveira E.H., Lopes J.A.D., Chaves M.H., Reis F.A.M., Citó A.M.G.L. (2008). Anacardic acid derivatives from Brazilian propolis and their antibacterial activity. Ecl. Quím. São Paulo.

[25] Rakotomanga M., Blanc S., Gaudin K., Chaminade P., Loiseau P.M. (2007). Miltefosine affects lipid metabolism in *Leishmania donovani* promastigotes. Antimicrob. Agents Chemother..

[26] Kozubek A., Tyman J.H. (1999). Resorcinolic lipids, the natural non-isoprenoid phenolic amphiphiles and their biological activity. Chem. Rev..

[27] Lee J.S., Cho Y.S., Park E.J., Kim J., Oh W.K., Lee H.S., Ahn J.S. (1998). Phospholipase Cγ1 inhibitory principles from the sarcotestas of Ginkgo biloba. J. Nat. Prod..

[28] Tanaka A., Araim Y., Kimm S., Hamm J., Usukim T. (2011). Synthesis and biological evaluation of bilobol and adipostatin A. J. Asian Nat. Prod. Res..

[29] Ebiloma G.U., Ayuga T.D., Balogun E.O., Gil L.A., Donachie A., Kaiser M., Herraiz T., Inaoka D.K., Shiba T., Harada S. (2018). Inhibition of trypanosome alternative oxidase without its N-terminal mitochondrial targeting signal (ΔMTS-TAO) by cationic and non-cationic 4-hydroxybenzoate and 4-alkoxybenzaldehyde derivatives active against *T. brucei* and *T. congolense*. Eur. J. Med. Chem..

[30] Alonezi S., Tusiimire J., Wallace J., Dufton M.J., Parkinson J.A., Young L.C., Clements C.J., Park J.K., Jeon J.W., Ferro V.A. (2016). Metabolomic profiling of the effects of melittin on cisplatin resistant and cisplatin sensitive ovarian cancer cells using mass spectrometry and biolog microarray technology. Metabolites.

[31] Sumner L.W., Amberg A., Barrett D., Beale M.H., Beger R., Daykin C.A., Fan T.W., Fiehn O., Goodacre R., Griffin J.L. (2007). Proposed minimum reporting standards for chemical analysis. Metabolomics.

