# Supplementary material for: Isolation of a Novel Flavanonol and an Alkylresorcinol with Highly Potent Anti-Trypanosomal Activity from Libyan Propolis
Source: Molecules. 2019 Mar 15;24(6):1041. doi: 10.3390/molecules24061041 (PMC6471328; doi:10.3390/molecules24061041)
Supplement: Supplementary file 1 [file molecules-24-01041-s001.pdf]

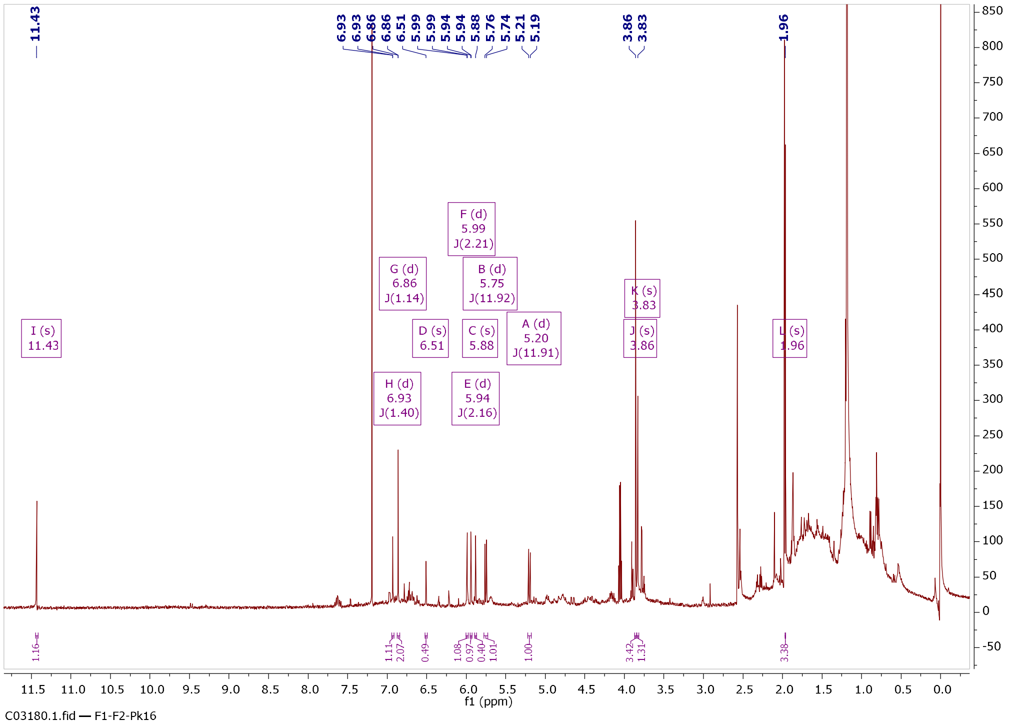

Figure S1 ^{1}H spectrum (600 MHz in $CDCl_3$) for compound **4**

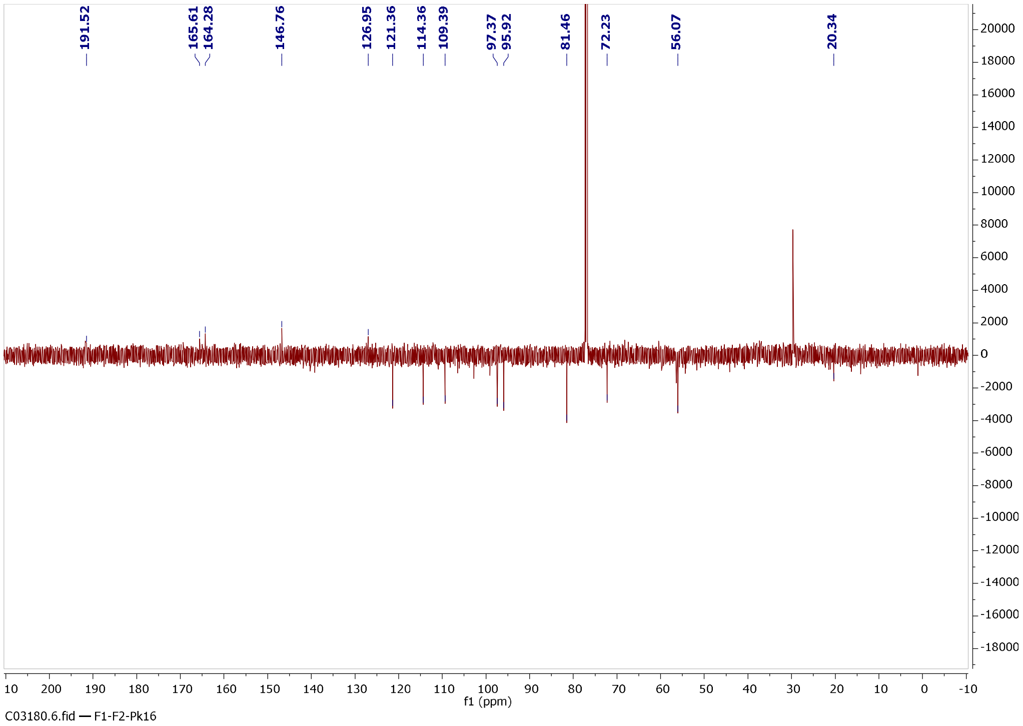

Figure S2 *J*-mod ^{13}C spectrum for compound **4**

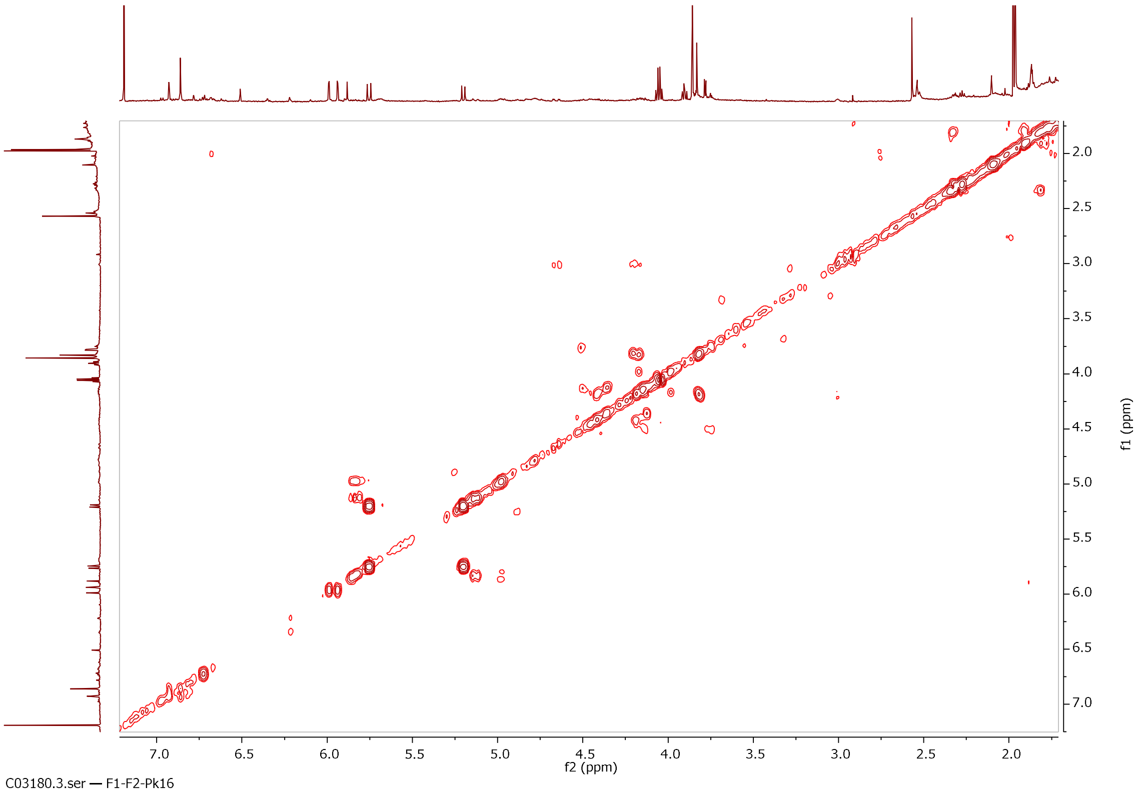

Figure S3 COSY spectrum for compound 4.

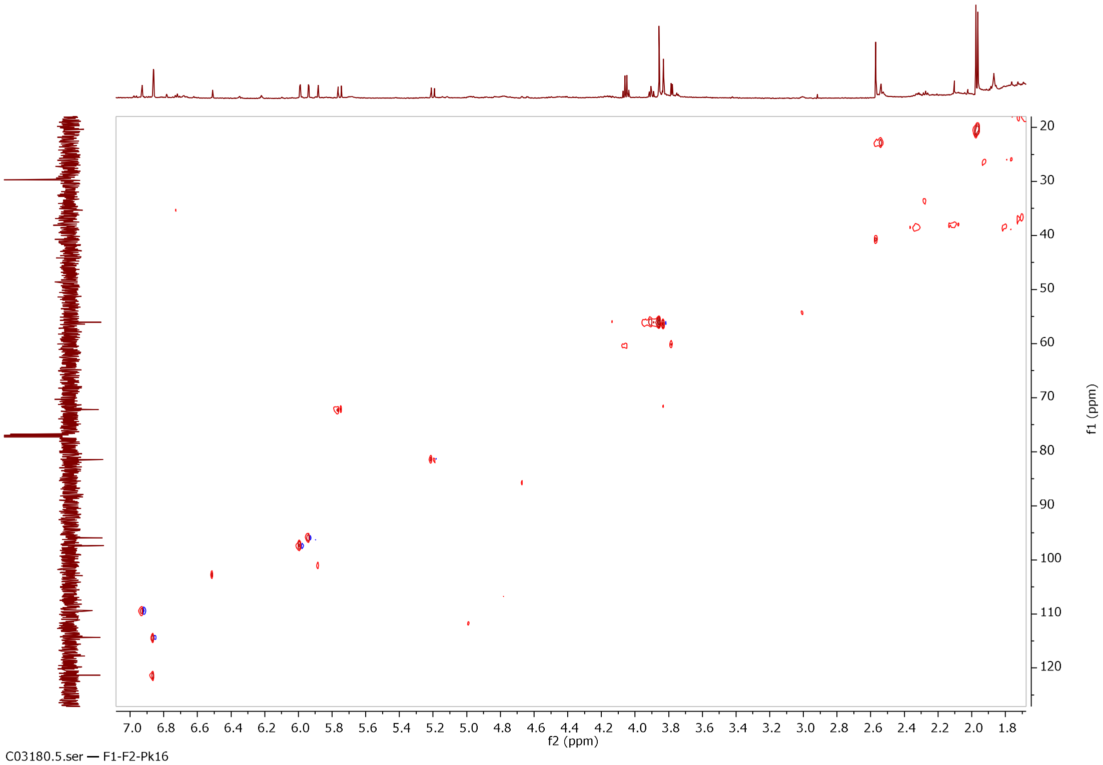

Figure S4 HSQC spectrum for compound **4**

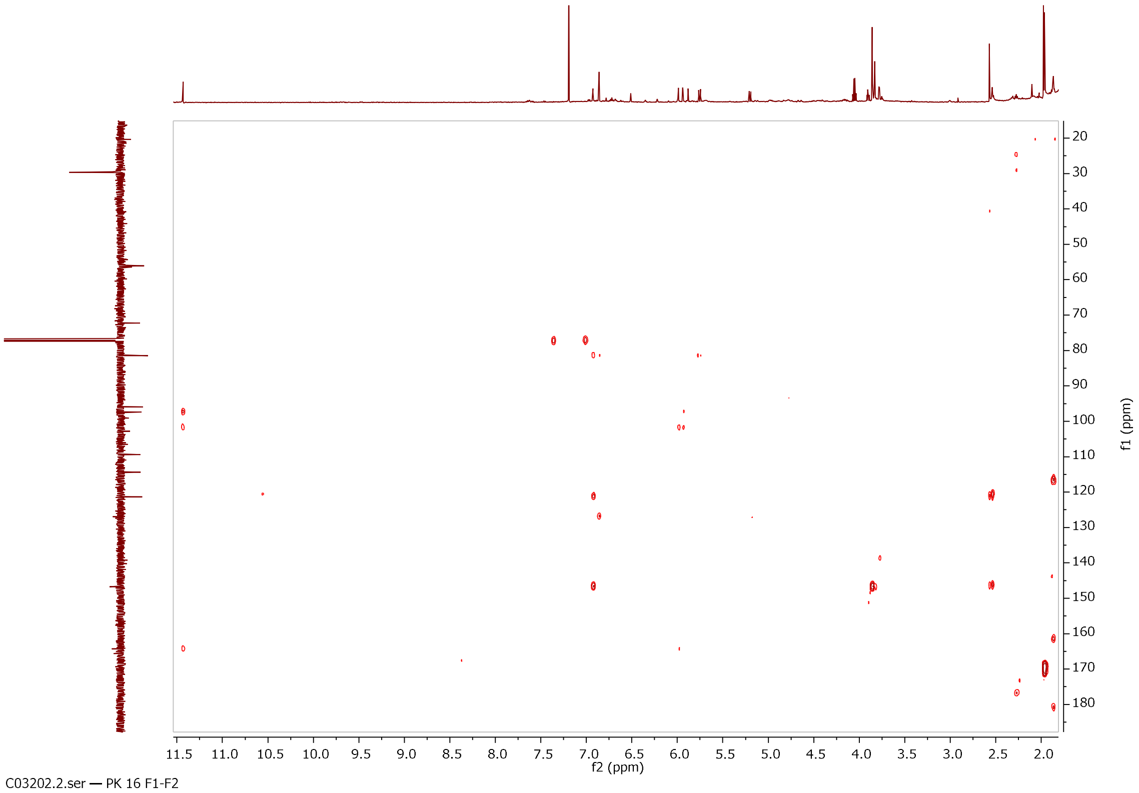

Figure S5 HMBC spectrum for compound **4**.

Table S1 ^{1}H and ^{13}C NMR of compound **4** (600 MHz, $CDCl_3$); Taxifolin-3-acetyl-4′-methylether

Position No	^{1}H δ (ppm)	^{13}C δ (ppm)
1	—	—
2	5.20 (1H, d, *J* = 11.7 Hz)	81.5
3	5.75 (1H, d, *J* = 11.7 Hz)	72.2
4	—	191.7
5	—	164.4
6	6.10 (1H, d, *J* =2.4 Hz)	97.4
7	-	164.4
8	6.05 (1H, d, *J* = 2.3 Hz)	95.8
9	—	163.1
10	—	101.8
1′	—	127.0
2′	6.86 (1H, m)	121.9
3′	—	146.8
4′	—	146.7
5′	6.93 (1H,m)	114.3
6′	6.99 (1H)	109.3
1″	—	169.2
4′-OCH_3	3.85 (3H, s)	56.13
2″-CH_3	1.96 (3H, s)	20.2
5-OH	11.52	—

Stevens et al. (1999) did not establish the position (3’ or 4’) of the O-methyl group but in the current study, by using 2D NMR, it was confirmed that compound **8** was Taxifolin-3-acetyl-4’-methyl ether as shown in Table S1 and thus can be considered to have been isolated for the first time.

[Stevens, J. F., Wollenweber, E., Ivancic, M., Hsu, V. L., Sundberg, S., & Deinzer, M. L. (1999). Leaf surface flavonoids of Chrysothamnus. *Phytochemistry*, *51*(6), 771-780.]

Table S2 ^1H and ^{13}C NMR of compound **1**; 13-epitorulosol compared to the literature[a]

No	^1H Experimental δ (ppm)	^{13}C Experimental δ (ppm)	^1H Literature[a] δ (ppm)	^{13}C Literature[a] δ (ppm)
1	1.80,1.06	39.0 (CH_2)	1.6,1.78 (m)	39.0 (CH_2)
2	1.51	19.0 (CH_2)	1.48 (m)	19.0 (CH_2)
3	1.05,1.51	35.7 (CH_2)	1.02,1.71(m)	35.4 (CH_2)
4	—	39.1 (C)	—	38.8 (C)
5	1.27	56.4 (CH)	1.22 (m)	56.3 (CH)
6	1.80 (m)<br>1.27 (m)	24.5 (CH_2)	1.29 (m)<br>1.82 (m)	24.4 (CH_2)
7	1.91 (m)<br>2.38 (m)	38.5 (CH_2)	1.92 (t d, J = 4, 12.5)<br>2.37 (t d, J = 4, 12.5)	38.6 (CH_2)
8	—	147.5 (C)	—	148.2 (C)
9	1.56 (m)	57.4 (CH)	1.54 (m)	57.3 (CH)
10	—	39.1 (C)	—	39.7 (C)
11	1.54,1.35	17.9 (CH_2)	1.36, 1.52 (2, m)	17.8 (CH_2)
12	1.26,1.73	41.3 (CH_2)	1.24, 1.76 (2, m)	41.3 (CH_2)
13	—	73.5 (C)	—	73.6 (C)
14	5.92 (dd, J = 17.4, 10.8)	145.2 (CH)	5.90 (dd, J = 10.8, 17.4)	145.1 (CH)
15	5.07 (J=10.8, 3)<br>5.12 (J=17.4, 1.33)	111.8 (CH_2)	5.04(d, J = 10.8)<br>5.19(d, J = 17.4)	111.6 (CH_2)
16	1.26	27.7 (CH_3)	1.26 (s)	27.1 (CH_3)
17	4.83<br>4.55	107.7 (CH_2)	4.51, 4.81 (s)	106.6 (CH_2)
18	1.00 (s)	27.2 (CH_3)	0.96 (s)	28.0 (CH_3)
19	3.74<br>3.37	65.8 (CH_2)	3.38, 3.74 (2d, J=10.9 each)	65.0 (CH_2)
20	0.67 (s)	15.4 (CH_3)	0.64 (s)	15.2 (CH_3)

[a]: Xue, J. J., Fan, C. Q., Dong, L., Yang, S. P., & Yue, J. M. (2004). Novel antibacterial diterpenoids from Larix chinensis Beissn. *Chemistry & Biodiversity*, *1*(11), 1702-1707.

Table S3 ^{1}H and ^{13}C NMR of compound **2**; demethylpiperitol (3, 4-Dihydroxy-3', 4'-methylenedioxy-7,9':7',9-diepoxylignan compared to the literature[a]

No	^{1}H Experimental δ (ppm)	^{13}C Experimental δ (ppm)	^{1}H Literature[a] δ (ppm)	^{13}C Literature[a] δ (ppm)
1	2.98	54.4	2.95 (m)	53.9
2	4.64	86.1	4.61 (d, 4.3)	85.0
3 (O)	–	–	–	–
4	3.80 (m)	71.6 (CH_2)	4.10 (dd, 6.8, 8.9)<br>3.69 (dd, 3.6, 9.1)	71.1
5	2.90 (m)	54.3 (CH)	2.95 (m)	53.6
6	4.64 (d, 12.4, 8.16)	85.6 (CH)	4.52 (d, 4.3)	84.8
7 (O)	–	–	–	–
8	3.80	71.9 (CH_2)	4.06 (dd, 6.8, 8.9)<br>3.71 (dd, 3.6, 9.1)	70.8
1'	–	135.0		135.6
2'	6.60 (1H, s)	102 (CH)	6.90 (d, 1.4)	106.6
3'	–	147.0	–	147.5
4'	5.5 (1H, s)	147.0	–	146.5
5'	6.77 (d, 8.0)	147.0	6.85 (d, 8.0)	147.4
6'	6.60 (1H, s)	102.3 (CH)	6.82 (dd, 1.4, 8.0)	119.4
1''	–	135.0	–	132.3
2''	6.88 (d, J = 1.5)	106.6 (CH)	6.71 (d, 2.0)	113.6
3''	–	147.9	–	145.1
4''	5.5 SA	147.1		144.7
5''	6.81 (d, J = 8)	108.4	6.66 (d, 8.0)	115.3
6''	6.83 (dd, J=1.5)	119.5	6.57 (dd, J=2.0, 8.0)	117.1
OCH2O	5.98 (2H,s)	101	5.97 (2H, s)	100.9
-OH	5.5 (1H,s)	147.0	5.84 (s)	146.8

[a]: Nakai, M., Harada, M., Nakahara, K., Akimoto, K., Shibata, H., Miki, W., & Kiso, Y. (2003). Novel antioxidative metabolites in rat liver with ingested sesamin. *Journal of Agricultural and Food Chemistry*, *51*(6), 1666-1670

Table S4 ^{1}H and ^{13}C NMR of compound **3**: 5'methoxypiperitol (1R,2S,5R,6S)-2-(3,4-methylenedioxyphenyl)-6-(4-hydroxy-3,5dimethoxyphenyl)-3,7-dioxabicyclo[3.3.0] octane compared to the literature[a]

No	^{1}H Experimental δ (ppm)	^{13}C Experimental δ (ppm)	^{1}H Literature δ (ppm)	^{13}C Literature δ (ppm)
1	2.98 (m)	54.3	3.07 (1H, m)	54.4
2	4.77 (m)	85.8	4.74 (1H, d, 4.9)	85.8
3 (O)	–	–	–	–
4	4.55<br>3.89	71.6	4.24 (1H, dd, 9.3, 6.8)<br>3.89 (1H, m)	71.9
5	3.12 (1H, m)		3.07 (1H, m)	54.3
6			4.70 (1H, d, 4.2)	86.1
7 (O)	–	–	–	–
8	4.36<br>3,77		4.27 (1H, dd, 9.0, 7.1)<br>3.87 (1H,m)	71.6
1′	–	135.2	–	135.1
2′	6.88 (d, J=1.53)	106.6	6.85 (1H,br s)	106.5
3′		147.1	–	147.1
4′		147.3	–	148.0
5′	6.80 (d, J = 8.0 Hz)	108.4	6.78 (1H, d, 8.0)	108.2
6′	6.83 (dd, J = 8.0, 1.5 Hz)	119.5	6.81 (1H, dd, 8.0, 1.2)	119.3
1′′		132.7	–	132.1
2′′	6.60 (1H, s)	102.7	6.58 (1H, s)	102.7
3′′		147.0	3.90 (3H, s)	147.1
4′′	5.50 (1H,s)	134.0	–	134.3
5′′		147.0	3.90 (3H, s)	147.1
6′′	6.60 (1H,s)	102.7	6.58 (1H, s)	102.7
OCH_2-O	5.98 (2H,s)	101.5	5.95 (2H, s)	101.1
OCH_3-3′′	3.93 (3H,s)	56.4	3.90 (3H, s)	56.4
OCH_3-5′′	3.93 (3H,s)	56.4	3.90 (3H, s)	56.4

[a]: Li, N., Wu, J. L., Hasegawa, T., Sakai, J. I., Bai, L. M., Wang, L. Y., ... & Tomida, A. (2007). Bioactive lignans from *Peperomia duclouxii*. *Journal of Natural Products*, *70*(4), 544-548

Table S5 ^{1}H and ^{13}C NMR of compound **5**; cycloartanol compared to the literature[a]

No	^{1}H Experimental δ (ppm)	^{13}C Experimental δ (ppm)	^{1}H Literature δ (ppm)	^{13}C Literature δ (ppm)
1	1.50 (m), 1.18 (m)	32.0	-	31.9
2	1.72, (m), 1.62 (m)	30.5	-	30.4
3	3.22 (m)	78.2	-	78.9
4	——	40.6	-	40.5
5	1.27	47.2	-	47.1
6	1.62 (m), 0.76 (m)	21.2	-	21.1
7	1.13 (m), 1.38 (m)	26.2	-	26.0
8	1.56	48.0	-	48.0
9	——	20.2	-	20.0
10	——	26.3	-	26.1
11	2.09, 1.12	26.6	-	26.6
12	1.75 (m)	33.1	-	32.9
13	——	45.5	-	45.2
14	——	48.91	-	48.7
15	1.43	35.7	-	35.9
16	1.48 (m), 2.08 (m)	28.2	-	28.1
17	1.76	52.4	-	52.3
18	1.13 (3H,s)	18.1	-	18.0
19	0.56 (d,4.1)<br>0.34 (d,4.3)	29.9	0.53(d,4.1)<br>0.32(d,4.3)	29.8
20	1.62	35.8	-	35.9
21	1.12	18.3	-	18.2
22	1.30, 1.73	36.4	-	36.3
23	2.18 (m), 2.30 (m)	25.0	-	24.9
24	5.12(obsc)	124.8	5.10 (obsc)	125.2
25	——	130.6	-	130.9
26	1.52 (1H, s)	17.2	-	17.6
27	1.70 (s)	25.7	-	25.7
28	1.03	19.5	-	19.3
29	1.14	25.5	-	25.4
30	0.99	14.04	-	14.0

[a]: Kardar, M. N., Zhang, T., Coxon, G. D., Watson, D. G., Fearnley, J., & Seidel, V. (2014). Characterisation of triterpenes and new phenolic lipids in Cameroonian propolis. *Phytochemistry*, *106*, 156-163.

Table S6 ^{1}H and ^{13}C NMR of compound **6**; mangiferolic compared to the literature[a]

No	^{1}H Experimental δ (ppm)	^{13}C Experimental δ (ppm)	^{1}H Literature δ (ppm)	^{13}C Literature δ (ppm)
1	1.57 (m),1.89 (m)	31.6	-	31.9
2	2.33, 2,73	29.7	-	30.3
3	3.32 (dd)	79.0	3.29 (m)	78.9
4	-	40.5	-	40.5
5	1.33	47.0	-	47.1
6	-	21.4	-	21.1
7	1.09,1.034	26.3	-	26.0
8	1.53 (dd)	47.9	-	47.9
9	-	20.1	-	19.9
10	-	26.1	-	26.0
11	1.91 (m), 1.16 (m)	26.4	-	26.4
12	1.66	32.1	-	32.9
13	-	45.4	-	45.3
14	-	48.8	-	48.8
15	1.3 (m)	35.6	-	35.5
16	1.92 (m)	28.4	-	28.1
17	1.61 (m)	52.2	-	52.2
18	1.0 (3H, s)	18.1	0.95(s)	18.1
19	0.53 (d,3.7), 0.35 (d, 6.0)	30.5	0.54 (d,3.7)<br>0.31 (d, 4)	29.9
20	1.44	36.0	-	36.0
21	0.93(d)	18.3	0.89 (d, 6.6)	18.1
22	1.55 (m), 1.16 (m),	33.7	-	34.7
23	2.23 (m), 2.20 (m)	26.2	-	25.9
24	6.93 (m)	145.8	6.89 (br t)	145.8
25	-	126.6	-	126.5
26	-	173.2	-	172.8
27	1.86 (s)	11.9	1.8 (s)	11.9
28	0.99 (s)	25.0	0.95 (s)	25.4
29	0.83 (s)	14.0	0.80 (s)	14.0
30	0.93 (s)	19.3	0.89 (s)	19.3

[a]: Kardar, M. N., Zhang, T., Coxon, G. D., Watson, D. G., Fearnley, J., & Seidel, V. (2014). Characterisation of triterpenes and new phenolic lipids in Cameroonian propolis. *Phytochemistry*, *106*, 156-163

Table S7 ^{1}H and ^{13}C NMR of compound **7**; mangiferonic acid compared to the literature[a]

No	^{1}H Experimental δ (ppm)	^{13}C Experimental δ (ppm)	^{1}H Literature δ (ppm)	^{13}C Literature δ (ppm)
1	188 (m),1.57 (m),	32.0		34.4
2	2.73 (dt,J=6.4, 13.9 Hz), 2.33 (m)	37.5	2.69(m) 2.29(m)	37.4
3	-	217.3	-	216.7
4	-	50.3	-	50.2
5	1.74 (m)	47.8	-	48.4
6	1.53 (m), 1.21 (m)	21.5	-	21.4
7	1.42 (m),1.17 (m)	25.9	-	25.9
8	1.62 (m)	47.9	-	47.8
9	-	21.1	-	21.0
10	-	25.9	-	25.9
11	2.10 (m), 1.14 (m)	26.2	-	26.6
12	1.77 (m)	32.8	-	32.7
13	-	45.4	-	45.4
14	-	48.8	-	48.7
15	1.32 (m)	35.6	-	35.5
16	1.92 (m)	28.2	-	28.1
17	1.62 (m)	51.6	-	52.2
18	1.02 (m)	17.6	0.98 (s)	18.1
19	0.77 (d,4.6) 0.56 (d4.2)	29.8	0.77 (d,3.7) 0.56 (d,3.8)	29.5
20	1.47 (m)	35.5	-	35.9
21	0.95 (s)	17.8	0.90 (br t,3.7)	18.1
22	1.23 (m),1.62 (m)	34.8	-	34.7
23	2.24 (m) ,2.40 (m)	25.9	-	25.8
24	7.01 (br t)	145.8	6.90 (br t,7.3)	145.7
25	-	126.6	-	126.6
26	-	171.9	-	172.8
27	1.85 (s)	11.9	1.83 (s)	12.0
28	1.07 (s)	22.2	1.05 (s)	22.1
29	1.12 (s)	20.78	1.10 (s)	20.8
30	0.93 (s)	18.3	0.90 (s)	19.2

[a]: Kardar, M. N., Zhang, T., Coxon, G. D., Watson, D. G., Fearnley, J., & Seidel, V. (2014). Characterisation of triterpenes and new phenolic lipids in Cameroonian propolis. *Phytochemistry*, *106*, 156-163

Table S8 ^{1}H and ^{13}C NMR of compound **8**; ambolic acid compared to the literature[a]

No	^{1}H Experimental δ (ppm)	^{13}C Experimental δ (ppm)	^{1}H Literature δ (ppm)	^{13}C Literature δ (ppm)
1	1.56 (m),124 (m)	31.9	-	31.9
2	1.76 (m),1.56 (m)	30.4	3.29 (m)	30.3
3	3.29 (m)	78.4	-	78.8
4	——	40.5	-	40.5
5	1.30 (m)	47.0	-	47.1
6	1.60 (m), 0.80 (m)	21.1	-	21.0
7	1.33 (m),1.08 (m)	26.2	-	26.0
8	1.51 (m)	47.9	-	48.0
9	——	19.9	-	20.0
10	——	26.2	-	26.1
11	1.99 (m), 1.13 (m)	26.5	-	26.4
12	1.53 (m, 2H)	32.9	-	32.9
13	——	45.4	-	45.3
14	——	48.8	-	48.8
15	1.28 (m)	35.5	-	35.5
16	1.78 (m),1.28 (m)	28.1	-	28.1
17	1.61 (m)	52.3	-	52.2
18	0.98 (3H, s)	18.1	0.95 (s)	18.0
19	0.56 (d, 4.1)<br>0.37 (d, 4.1)	29.8	5.4 (d, 4)<br>0.33 (d, 4)	29.9
20	1.31 (m)	36.1	-	36.0
21	0.89 (d, 6.5, 3H)	18.4	0.89 (d, 6.6)	18.3
22	1.55 (m),1.21 (m),	34.6	-	34.5
23	2.12 (m),1.95 (m)	26.1	-	25.9
24	——	148.8	-	148.7
25	3.14 (br m)	45.5	3.16 (br q, 6.6)	45.6
26	——	179.9	-	179.8
27	1.27 (d,6.5)	16.1	1.29 (d, 7.1)	16.3
28	——	25.5	0.95 (s)	25.4
29	0.85 (s, 3H)	14.2	0.80 (s)	14.0
30	0.89 (s, 3H)	19.4	0.89 (s)	19.3
31	4.98 (br s)<br>4.94 (br s)	111.1	4.96 (br s)<br>4.92 (br s)	111.0

[a]: Kardar, M. N., Zhang, T., Coxon, G. D., Watson, D. G., Fearnley, J., & Seidel, V. (2014). Characterisation of triterpenes and new phenolic lipids in Cameroonian propolis. *Phytochemistry*, *106*, 156-163

Table S9 ^{1}H and ^{13}C NMR of compound **9**; 27-Hydroxymangiferonic acid compared to the literature[a]

No	^{1}H Experimental δ (ppm)	^{13}C Experimental δ (ppm)	^{1}H Literature δ (ppm)	^{13}C Literature δ (ppm)
1	1.89 (m), 1.57 (m)	32.9	1.89 (m),1.54 (m)	33.4
2	2.73 (dt,J=6.4,13.9 Hz)<br>2.33 (m)	36.9	2.71 (m), 2.31 (m)	37.2
3	-	216.7	-	216.6
4	-	49.7	-	50.2
5	1.74 m	48.2	1.71 (dd)	48.4
6	1.55 (m),0.89 (m)	21.4	1.57 (m), 0.95 (m)	21.5
7	1.33 (m),1.02 (m)	26.2	1.38 (m),1.14 (m)	25.9
8	1.62 (m)	47.6	1.59 (m)	47.8
9	-	21.0	-	21.2
10	-	25.9	-	26.00
11	189 (m),1.22 (m)	26.2	1.38 (m),1.14 (m)	26.8
12	1.65 (m)	32.7	1.61 (m)	32.8
13	-	45.4	-	45.4
14	-	48.7	-	48.8
15	1.30	35.5	1.32 (m, 2H)	35.6
16		28.4	1.92 (m)	28.2
17		51.6	1.61 (m)	52.2
18	1.02 (3H,s)	17.8	1.00 (3H,s)	18.12
19	0.77 (d,4.2)<br>0.56 (d,4.2)	29.5	0.79 (d,4.2)<br>0.58 (d,4.2)	29.6
20	1.47	35.9	1.44 (m)	36.00
21	0.94 (3H,s)	17.6 (CH_3)	0.92 (d,6.,3H)	18.2
22	1.23 (m) ,1.62 (m)	29.2	1.58 (m), 1.17 (m)	34.8
23	2.24 (m), 2.40 (m)	25.8	2.26 (m), 2.12 (m)	25.9
24	7.04	146.5	6.91 (br t)	145.80
25	-	129.3	-	126.6
26	-	171.1	-	173.1
27	4.38 (s)	56.6	4.22 (s)	57.2
28	0.93 (3H,s)	22.1	1.05 (3H, s)	22.2
29	1.07 (s)	20.8	1.02 (3H, s)	20.7
30	0.93 (s)	18.8	0.91 (3H, s)	19.3

[a]: Anjaneyulu, V., & Radhika, P. (2000). The triterpenoids and steroids from *Mangifera indica* Linn. *Indian Journal of Chemistry*, *39B*, 883- 893.

Table S10 ^{1}H and ^{13}C NMR of cardol (**10**) in a partially purified fraction.

No	^{1}H Experimental δ (ppm)	^{13}C Experimental δ (ppm)	^{1}H Literature* δ (ppm)	^{13}C Literature* δ (ppm)
1	-	156.8		156.6
2	6.19 t (J = 2.1)	100.3	5.15	100.2
3	-	156.8		156.6
4	6.24 d (J = 2.1)	107.9	6.22	108.1
5	-	146.0		146.2
6	6.24 d (J = 2.1)	107.9	6.22	108.1
1′	2.45 brt (J = 7.8)	35.9	2.54	35.9
2′	1.53 m	31.1		
3′	1.32 m	29.4		
4′-6′	1.28-1.37 env			
7′	2.02 m	27.2		
8′	5.35m	129.9	5.33	129.9
9′	5.35 m	129.88	5.33	129.9
10′	2.02 m	26.9		
11′-13′	1.28-1.37 env	32.1		31.7
14′	1.33 m	22.4		22.5
15′	0.90	14.0		14.1

*Silva et al (2008)

Compound **10** is a partially purified fraction with estimated (from ^{1}H NMR spectrum) 60% of 5-(8Z-pentadecenyl)-benzene-1,3-diol with mangiferolic acid (30%) and 10% anacardic acid. The proton NMR spectrum of the fraction showed that the major compound had aromatic signals that were quite shielded, with a 1H triplet (J = 2.1Hz) at δ6.22 ppm coupled to a 2-proton doublet at δ6.26 ppm indicating a tri-substituted symmetrical ring. The ^{13}C NMR and HSQC confirmed this and the presence of two phenolic hydroxyls with equivalent carbons at 156.8 ppm, along with two other equivalent CH carbons at 107.9 that were directly coupled with the δ 6.26 proton pair of a resorcinol-type compound.

In the HMBC spectrum the proton at δ 6.26 ppm coupled with a methylene carbon at 35.9 ppm (which had a ^{1}J coupling from the broad proton triplet at δ2.48 in the HSQC), a carbon at 100.3 ppm and to “its own carbon” at 107.9 ppm giving further indication of symmetry in that part of the compound. Additionally, the methylene protons at δ2.48 ppm showed long-range correlations to the aromatic carbons C-4/6 (107.9 ppm) and C-5 (146.0 ppm) and two methylenes C-2’ (31.1 ppm) and C-3’ (29.4 ppm) bearing the protons at δ1.57 ppm and δ1.34 ppm, respectively. There were also two olefinic protons, both appeared at δ5.35 ppm attached to the olefinic carbons at 129.88 and 129.9 ppm and two adjacent methylenes (COSY) at δ2.04 ppm (attached to the carbons at 26.9 and 27.2 ppm). A methylene envelope in the ^{1}H NMR between δ1.30-1.40 ppm and several overlapping carbon signals between 29.0 and 30.0 ppm in the ^{13}C spectrum did not give the precise overall length of the chain but the LC-MS gave a molecular ion at 318 amu with fragmentation in MS-MS suggesting an 8’-position for one *cis* double bond on a 15-carbon side chain.

Thus the major substance in the fraction was an alkyl resorcinol derivative 5-(8Z-pentadecenyl)-benzene-1,3-diol also known as bilobol (Cirigotis & Cleaver, 1974; Lytollis et al., 1995; Lee et al., 1998). The crude mixtures were shown to have anti-bacterial activity against *Escherichia coli*, *Staphylococcus aureus*, *Pseudomonas aeruginosa* and *Shigella* spp.

Park et al. (2009) took a cardol mixture from cashew nut shell liquid (CNSL) as a renewable substance to enzymatically synthesize polycardol polymers. They estimated that the polymer prepared contained 10% of 5-(8Z-pentadecenyl)-benzene-1,3-diol derived from the cashew liquid. It is interesting that bees would select 5-(8Z-pentadecenyl)-benzene-1,3-diol type compounds to incorporate into their propolis when the above authors are attempting to produce thermostable polymers using the same CNSL starting materials.

References

Cirigottis K., & Cleaver L.E.A. (1974) Chemical Studies of the Proteaceae. Vii. An examination of the woods of 17 species for resorcinol derivatives. *Australian Journal of Chemistry*, *27*(2). 345 - 355.

Lee, J.S., Cho, Y.S., Park, E.J., Kim, J., Oh, W.K., Lee H.S., & Ahn, J.S. (1998) Phospholipase Cγ1 Inhibitory Principles from the Sarcotestas of *Ginkgo biloba*. *Journal of Natural Products 61*(7), 867-871.

Lytollis, W., Scannell, R.T., An, H., Murty, V.S., Reddy, S.,Barr, J.R., & Hecht S.M. (1995) 5-Alkylresorcinols from *Hakea trifurcata* that cleave DNA. *Journal of the American Chemical Society*, *117*, 12683-12690.

Park, S.Y., Kim, Y.H., Won, K., & Song BK (2009) Enzymatic synthesis and curing of polycardol from renewable resources. *Journal of Molecular Catalysis B: Enzymatic*, *57*(1-4), 312-316.

Silva, M. S. S., De Lima. S. G., Oliveira, E. H, Lopes, J. A. D., Chaves, M. H., Reis, F. A. M., Citó, A. M. G. L. (2008). Anacardic acid derivatives from Brazilian propolis and their antibacterial activity. *Ecl. Quím., São Paulo*, *33*(3): 53-58.

Table S11 ^{1}H and ^{13}C NMR of compound **11** (400 MHz, $CDCl_3$); Acetylisocuppressic acid compared to the literature[a]

No	^{1}H Experimental δ (ppm)	^{13}C Experimental δ (ppm)	^{1}H Literature δ (ppm)[a]	^{13}C Literature δ (ppm)[a]
1	0.99 (m), 1.66 (m)	39.4	0.98 (m), 1.76 (m)	39.1
2	1.35 (m).1.77 (m)	20.0	1.46 (m) 1.77 (m)	19.9
3	2.42 (m)	38.0	2.10 (m)	37.9
4	-	44.1	-	44.2
5	1.28	56.3	1.32	56.3
6	(m)	25.6	1.79 (m)	26.1
7	2.43 (m)	38.7	1.77 (m)	38.7
8	-	147.9	-	147.9
9	1.53 (m)	55.5	1.49 (m)	55.4
10	-	40.4	-	40.4
11	1.45 (m),1.62 (m)	21.8	1.38 (m),1.56 (m)	21.8
12	1.84 (m), 2.16	38.4	0.98 (m),1.81 (m)	38.4
13	-	142.9	-	143.0
14	5.28 (t)	118.1	5.23tr	118.0
15	4.56 (d)	61.4	4.51 (d, 7.1)	61.5
16	1.66 (s)	16.6	1.62(s)	16.6
17	4.49 (br s),4.83 (br s)	106.5	4.45 (br s),4.78 (br s)	106.5
18	1.23 (s)	28.9	1.17s	29.0
19	-	182.4	-	183.5
20	0.57 (s)	12.8	0.53 (s)	12.8
21	-	171.4	-	171.2
22	2.03 (s)	21.4	2.23 (s)	21.1

[a]: Popova, M. P., Chinou, I. B., Marekov, I. N., & Bankova, V. S. (2009). Terpenes with antimicrobial activity from Cretan propolis. *Phytochemistry*, *70*(10), 1262-1271.

By comparison, of ^{1}H and ^{13}C NMR spectra of compound **12** with the literature it was identified as agathadiol.

Table S12 ^{1}H and ^{13}C NMR of compound **12** (400 MHz, $CDCl_3$), agathadiol compared to the literature[a]

C	^{1}H Experimental δ (ppm)	^{13}C Experimental δ (ppm)	^{1}H Literature δ (ppm) [a]	^{13}C Literature δ (ppm) [a]
1	1.01 (m)	38.6 (CH_2)	0.98m,176	38.5
2	1.51 (m)	19.0 (CH_2)	1.46m,177	19.1
3	1.05, 1.51	35.5 (CH_2)	2.10 m	35.6
4	-	38.8 (C)	-	39.6
5	0.96	56.3 (CH)	1.32	56.4
6	1.80, 1.27	24.1 (CH)	1.79m	24.6
7	2.38, 1.91	38.7 (CH_2)	1.77m, 2.33m	39.2
8	-	147.5 (CH)	-	148.2
9	1.56	56.3 (CH)	1.49m	56.6
10	-	40.00 (C)	-	38.9.6
11	1.54, 1.35	17.9 (CH_2)	1.38m,1.56m	22.1
12	2.16, 2,39	38.4 (CH_2)		38.7
13	—	140.6 (C)		140.3
14	5.39 (t)	123.2	*5.39 t(6.9)*	123.3
15	4.16 (d, *J* =12)	59.5	4.15 d(6.9)	59.4
16	1.68 (br s)	17.00	1.67 (br s)	16.3
17	4.80 (br s)<br>4.51 (br s)	107.7	4.59 (br s)<br>4.92 (br s)	106.6
18	0.67 (s)	15.4	0.52 (3H, s)	27.2
19	3.75 (d, J=10.8)<br>3.40 (dd, 10.8, 1)	65.1 (CH_2)	1.16 (3H, s)	65.1
20	0.65 (s)	19.0 (CH_2)	0.65 (s)	18.3

[a]: San Feliciano, A., Medarde, M., Lopez, J. L., Del Corral, J. M., Puebla, P., & Barrero, A. F. (1988). Terpenoids from leaves of *Juniperus thurifera*. *Phytochemistry*, *27*(7), 2241-2248

Compound **13** had the elemental composition $C_{20}H_{32}O_3$. It had an exomethylenegroup, a carboxylic acid and propyl alcohol group. By comparison of ^1H and ^{13}C NMR spectra of compound **(17)** with the literature it was identified as isocupressic acid (ent-labd-18(17), 13-*E*-dien-15-ol-18-oic acid).

Table S13 ^1H and ^{13}C NMR of compound **13** (400 MHz, $CDCl_3$); (isocupressic acid[a]) compared to the literature[a]

No	^1H Experimental δ (ppm)	^{13}C Experimental δ (ppm)	^1H Literature δ (ppm)[a]	^{13}C Literature δ (ppm)[a]
1	1.01 (m)	38.6 (CH_2)	-	39.1
2	1.51 (m)	19.0 (CH_2)	-	19.3
3	1.05, 1.51	36.9 (CH_2)	-	37.9
4	-	44.7	-	44.2
5	0.96 (s)	56.3 (CH)	-	56.3
6	1.80, 1.27	25.6 (CH)	-	26.1
7	2.38, 1.91	38.7 (CH_2)	-	38.4
8	-	147.5 (CH)	-	147.9
9	1.56	56.3	-	55.5
10	-	40.00	-	40.4
11	1.54, 1.35	17.9 (CH_2)	-	24.1
12	2.16, 2,39	38.4	-	38.7
13	-	140.6	-	140.5
14	5.39 (br t)	123.2	5.40 (t, J = 7)	122.9
15	4.16 (d, 7)	59.5	4.87 (s,1H)	59.3
16	1.67 (br s)	17.00	1.67 (3H, s)	16.3
17	4.86 (br s)<br>4.51 (br s)	107.7	4.17 (d, J = 7, 2H),<br>4,54 (s, 1H)	106.5
18	3.74,3.337	29.0	1.25(3H s)	29.0
19	-	183.3	-	183.6
20	1.15 (s)	13.9	0.62 (3H s),	13.6

[a]: Stegelmeier, B. L., Ralphs, M. H., Gardner, D. R., Molyneux, R. J., & Jame, L. F. (1994). Serum α-mannosidase activity and the clinicopathologic alterations of locoweed (Astragalus mollissimus) intoxication in range cattle. *Journal of Veterinary Diagnostic Investigation*, *6*(4), 473-479.

Compound **14** is an isomer of compound **1** with the OH group shifted from position 15 to 13 and the double bond from 13, 14 to 14, 15 Table 3-53. By comparison, of ^{1}H and ^{13}C NMR spectra of compound **14** with the literature it was identified as isoagatholal.

Table S14 ^{1}H and ^{13}C NMR of compound **14** in (400 MHz, $CDCl_3$); Isoagatholal compared to the literature[a]

**No**	**^{1}H Experimental δ (ppm)**	**^{13}C Experimental δ (ppm)**	**^{1}H Literature δ (ppm)**	**^{13}C Literature δ (ppm)**
1	1.05 (m)	38.4	-	38.4
2	1.41 (m)	19.4	-	19.3
3	1.01, 1.51	34.4	-	38.
4	—	48.7	-	48.6
5	0.98 (s)	56.1	-	55.0
6	1.80 (m), 1.27 (m)	22.9	-	22.1
7	2.38, 1.91	38.5	-	38.1
8	—		-	147.3
9	1.63	54.9	2.44 (m)	56.1
10	—	40.1	-	40.1
11	1.51 (m), 1.27 (m)		-	24.1
12	2.16 (m), 2,39 (m)	38.5	-	38.5
13	—	140.3	-	139.7
14	5.38	123.3	5.35 (t, J = 6.8)	123.5
15	4.16 (J = 6.8)	59.5	4.11 (d, J = 6.8)	59.2
16	1.67 (3H, s)	16.4	1.65 (3H,s)	16.3
17	4.89 (s), 4.51 (s)	107.3	4.86 (s),4.53 (s)	107.3
18	1.02 (3H, s)	24.4	1.02 (3H,s)	24.4
19	9.74 (s)	205.9(CH)	9.70 (s)	205.5
20	0.57 (3H, s)	13.8	0.57 (s)	13.6

[a]: Hasegawa, S., & Hirose, Y. (1980). A diterpene glycoside and lignans from seed of *Thujopsis dolabrata*. *Phytochemistry*, *19*(11), 2479-2481.

Table S15 Significant metabolite changes produced by cardol in *T. brucei* treatment control. Positive ion data matched to within 3 ppm of exact mass. * Matches retention time of standard.

Row m/z	Row retention time	Molecular formula	Name	p-value 1/5	Ratio 1/5
489.1144	15.0	$C_{14}H_{26}N_4O_{11}P_2$	CDP-choline	<0.001	3.023
756.5539	4.0	$C_{42}H_{78}NO_8P$	PC 34:3	0.001	0.285
828.5541	3.8	$C_{48}H_{78}NO_8P$	PC40:9	0.001	0.190
174.0874	14.2	$C_6H_{11}N_3O_3$	5-Guanidino-2-oxopentanoate	0.002	2.408
222.0969	11.6*	$C_8H_{15}NO_6$	N-Acetyl-D-glucosamine	0.002	3.618
792.5746	3.7	$C_{42}H_{82}NO_{10}P$	PS36:0	0.005	0.325
204.1229	10.9	$C_9H_{18}NO_4$	O-Acetylcarnitine	0.008	0.394
804.5534	3.8	$C_{46}H_{78}NO_8P$	PC38:7	0.009	0.413
758.5694	3.9	$C_{42}H_{80}NO_8P$	PC34:2	0.010	0.424
780.5538	3.9	$C_{44}H_{78}NO_8P$	PC36:5	0.010	0.374
166.0862	10.1*	$C_9H_{11}NO_2$	Phenylalanine	0.010	0.676
104.1069	20.3*	$C_5H_{13}NO$	Choline	0.012	2.095
814.5596	3.5	$C_{44}H_{80}NO_{10}P$	PS38:3	0.012	0.360
161.1283	23.8*	$C_7H_{16}N_2O_2$	Methyllysine	0.012	3.718
854.5689	3.8	$C_{50}H_{80}NO_8P$	PC42:10	0.013	0.428
782.5699	3.9	$C_{44}H_{80}NO_8P$	PC36:4	0.013	0.445
216.0629	15.4*	$C_5H_{14}NO_6P$	Glycero phosphoethanolamine	0.015	0.684
716.5234	3.8	$C_{39}H_{74}NO_8P$	PE34:2	0.015	0.025
796.5857	3.9	$C_{45}H_{82}NO_8P$	PE40:4	0.017	0.332
261.0366	15.5*	$C_6H_{13}O_9P$	Glucose 6-phosphate	0.017	1.255
858.5999	3.8	$C_{50}H_{84}NO_8P$	PC42:8	0.017	0.449
840.5749	3.5	$C_{46}H_{82}NO_{10}P$	PS40:4	0.018	0.402
129.0658	14.7	$C_5H_8N_2O_2$	Dihydrothymine	0.019	1.400
856.5847	3.8	$C_{50}H_{82}NO_8P$	PC42:9	0.020	0.472
744.5563	3.8	$C_{41}H_{78}NO_8P$	PC36:2	0.022	0.028
842.5903	3.5	$C_{46}H_{84}NO_{10}P$	PS40:3	0.023	0.440
760.5852	3.9	$C_{42}H_{82}NO_8P$	PC38:1	0.025	0.492
133.097	23.3*	$C_5H_{12}N_2O_2$	Ornithine	0.025	1.440
260.0526	16.5	$C_6H_{14}NO_8P$	Glucosamine 6-phosphate	0.031	2.776
175.1188	26.6*	$C_6H_{14}N_4O_2$	Arginine	0.031	1.485
335.0734	15.6	$C_9H_{19}O_{11}P$	sn-glycero-3-Phospho-1-inositol	0.034	0.708
844.6058	3.5	$C_{46}H_{86}NO_{10}P$	PS40:2	0.035	0.444
175.1439	21.6	$C_8H_{18}N_2O_2$	Dimethyllysine	0.035	2.007
664.1164	13.9*	$C_{21}H_{28}N_7O_{14}P_2$	NAD+	0.036	1.227
882.6	3.8	$C_{52}H_{84}NO_8P$	PC44:10	0.039	0.432
816.5749	3.5	$C_{44}H_{82}NO_{10}P$	PS38:2	0.043	0.374
146.0924	15.0	$C_5H_{11}N3O_2$	4-Guanidinobutanoate	0.045	7.174
204.0864	11.6	$C_8H_{13}NO_5$	Acetyl-L-aminoadipate	0.048	2.586

124.0392	7.4*	$C_6H_5NO_2$	Nicotinate	0.050	3.204
860.6159	3.8	$C_{50}H_{86}NO_8P$	PC42:7	0.050	0.419
830.5694	3.8	$C_{48}H_{80}NO_8P$	PC 40:8	0.087	0.600
832.5847	3.8	$C_{48}H_{82}NO_8P$	PC40:7	0.097	0.609

Table S16 Appendix Metabolites in negative ion in cells cardol treated versus control.

Row m/z	Row retention time	Molecular formula	Name	p-value 1/5	Ratio 1/5
214.0489	15.4	$C_5H_{14}NO_6P$	sn-glycero-3-Phosphoethanolamine	<0.01	0.74
165.0558	4.9	$C_9H_{10}O_3$	3-(3-Hydroxy-phenyl)-propanoic acid	<0.01	0.68
471.1059	13.9	$C_{14}H_{27}N_4O_{10}P_2$	CMP-N-trimethyl-2-aminoethylphosphonate	<0.01	2.11
487.1009	15.0	$C_{14}H_{26}N_4O_{11}P_2$	CDP-choline	<0.01	2.88
606.0755	14.6	$C_{17}H_{27}N_3O_{17}P_2$	UDP-N-acetyl-D-glucosamine	<0.01	2.47
300.0495	14.6	$C_8H_{16}NO_9P$	N-Acetyl-D-glucosamine 6-phosphate	<0.01	1.78
146.0459	10.6	$C_5H_9NO_4$	Glutamate	<0.01	0.65
140.012	15.4	$C_2H_8NO_4P$	Ethanolamine phosphate	<0.01	0.71
159.03	14.0	$C_6H_8O_5$	2-Oxoadipate	<0.01	1.79
132.0303	14.6	$C_4H_7NO_4$	Aspartate	<0.01	0.38
769.5028	3.5	$C_{42}H_{75}O_{10}P$	PG36:4	<0.01	0.25
131.0827	26.6	$C_5H_{12}N_2O_2$	Ornithine	<0.01	1.56
173.1045	26.6	$C_6H_{14}N_4O_2$	Arginine	<0.01	1.48
173.0933	13.3	$C_7H_{14}N_2O_3$	N-Acetylornithine	<0.01	1.47
164.0718	10.1	$C_9H_{11}NO_2$	Phenylalanine	<0.01	0.67
695.4666	3.6	$C_{39}H_{69}O_8P$	GP 36:4	<0.01	0.08
687.5458	4.1	$C_{38}H_{77}N_2O_6P$	SP32:0	0.01	0.28
253.2178	3.8	$C_{16}H_{30}O_2$	Hexadecenoic acid	0.01	0.58
155.0462	10.8	$C_6H_8N_2O_3$	Imidazolonepropanoate	0.01	1.55
857.5187	3.6	$C_{45}H_{79}O_{13}P$	PI36:4	0.01	0.30
738.5089	3.9	$C_{41}H_{74}NO_8P$	PE36:4	0.01	0.15
808.1185	11.9	$C_{23}H_{38}N_7O_{17}P_3S$	Acetyl-CoA	0.01	0.43
671.467	3.6	$C_{37}H_{69}O_8P$	GP34:2	0.01	0.37
195.0512	13.5	$C_6H_{12}O_7$	D-Gluconic acid	0.01	0.85
131.0827	23.3	$C_5H_{12}N_2O_2$	L-Ornithine	0.01	1.66
821.5349	3.5	$C_{46}H_{79}O_{10}P$	PG 40:6	0.01	0.34
422.2308	3.6	$C_{19}H_{38}NO_7P$	LysoPE 14:1	0.01	0.52
213.0172	14.3	$C_5H_{11}O_7P$	Deoxyribose phosphate	0.01	0.72
243.0625	11.8	$C_9H_{12}N_2O_6$	Uridine	0.01	0.81
429.3378	3.6	$C_{28}H_{46}O_3$	Cholestatriene diol	0.01	7.73
662.1028	13.8	$C_{21}H_{28}N_7O_{14}P_2$	NAD+	0.01	1.14
333.0599	15.7	$C_9H_{19}O_{11}P$	Glycero-3-Phospho-1-inositol	0.01	0.73
455.3539	4.0	$C_{30}H_{48}O_3$	Ursolic acid	0.02	51.16
133.0143	15.4	$C_4H_6O_5$	Malate	0.02	0.50
111.02	9.6	$C_4H_4N_2O_2$	Uracil	0.02	2.04
481.9779	17.7	$C_9H_{16}N_3O_{14}P_3$	CTP	0.02	3.07
835.5338	3.6	$C_{43}H_{81}O_{13}P$	PI 34:1	0.02	0.49
823.55	3.5	$C_{46}H_{81}O_{10}P$	PG 40:5	0.02	0.45
96.96972	12.4	H_3O_4P	Orthophosphate	0.02	0.74

134.0471	9.7	$C_5H_5N_5$	Adenine	0.02	1.94
786.5299	3.6	$C_{42}H_{78}NO_{10}P$	PS36:2	0.02	0.46
445.0541	16.0	$C_{11}H_{20}N_4O_{11}P_2$	CDP-ethanolamine	0.02	1.94
352.9687	16.8	$C_6H_{12}O_{13}P_2$	Arabinitol bisphosphate	0.02	2.36
442.0178	17.4	$C_{10}H_{15}N_5O_{11}P_2$	GDP	0.02	1.25
118.051	14.1	$C_4H_9NO_3$	Threonine	0.03	0.79
401.0163	14.0	$C_{10}H_{16}N_2O_{11}P_2$	dTDP	0.03	3.40
253.0712	17.2	$C_6H_{15}N_4O_5P$	Arginine phosphate	0.03	0.23
259.0227	15.5	$C_6H_{13}O_9P$	Glucose phosphate	0.03	1.27
308.9788	16.1	$C_5H_{12}O_{11}P_2$	Ribose-bisphosphate	0.03	1.95
453.3383	3.9	$C_{30}H_{46}O_3$	Cholestapentaene-1,3,25-triol	0.03	904.73
158.0461	12.8	$C_6H_9NO_4$	4-Methylene-L-glutamate	0.03	0.88
241.0125	16.2	$C_6H_{11}O_8P$	Inositol 1,2-cyclic phosphate	0.03	1.59
347.0403	14.9	$C_{10}H_{13}N4O_8P$	IMP	0.03	0.41
199.0015	12.4	$C_4H_9O_7P$	Erythrose 4-phosphate	0.03	0.67
825.5656	3.5	$C_{46}H_{83}O_{10}P$	PG40:4	0.03	0.47
124.0074	14.6	$C_2H_7NO_3S$	Taurine	0.03	0.84
373.2753	3.6	$C_{24}H_{38}O_3$	3-Oxo-5beta-cholanate	0.03	44.46
111.02	8.5	$C_4H_4N_2O_2$	Uracil	0.03	1.93
345.244	3.6	$C_{22}H_{34}O_3$	Hydroxydocosapentaenoic acid	0.03	85.93
369.2439	3.6	$C_{24}H_{34}O_3$	Oxocholadienoic acid	0.04	#DIV/0!
419.3177	4.1	$C_{26}H_{44}O_4$	Cholestadiene tetrol	0.04	1684.97
96.96022	17.3	H_2O_4S	Sulfate	0.04	1.06
375.291	3.6	$C_{24}H_{40}O_3$	Hydroxycholanate	0.04	47.64
426.0231	14.7	$C_{10}H_{15}N_5O_{10}P_2$	ADP	0.04	1.63
401.3067	3.6	$C_{26}H_{42}O_3$	Cholestatriene diol	0.04	143.94
402.9957	15.9	$C_9H_{14}N_2O_{12}P_2$	UDP	0.04	1.60
833.5189	3.6	$C_{43}H_{79}O_{13}P$	PI 34:2	0.05	0.62
166.9753	12.4	$C_3H_5O_6P$	Phosphoenolpyruvate	0.05	0.65
245.0434	12.3	$C_6H_{15}O_8P$	Glycerophosphoglycerol	0.05	0.91
467.3537	3.8	$C_{31}H_{48}O_3$	Hydroxy-vitamin K	0.05	69.34

Table S17 Negative ion data for the supernatants of from treated versus control negative ion data matched to within 3 ppm of exact mass.* Retention time matches that of authentic standard.

Row m/z	Row retention time	Molecular formula	Name	p-value S1/S5	Ratio S1/S5
369.2439	3.6	$C_{24}H_{34}O_3$	Oxochola-4,6-dien-24-oic Acid	<0.001	>10000
455.3539	4.0	$C_{30}H_{48}O_3$	Ursolic acid	<0.001	618.469
345.244	3.6	$C_{22}H_{34}O_3$	Hydroxydocosapentaenoic acid	0.006	735.070
221.0604	16.8*	$C_7H_{14}N_2O_4S$	Cystathionine	0.009	68.368
616.4721	4.2	$C_{34}H_{68}NO_6P$	hexadecanoyl-sphineninephosphate	0.010	18.225
115.0401	4.0	$C_5H_8O_3$	Oxopentanoic acid	0.014	0.811
243.1974	3.9	$C_{14}H_{28}O_3$	Hydroxytetradecanoic acid	0.015	2.40
695.4666	3.6	$C_{39}H_{69}O_8P$	GP36:4	0.015	16.22
331.2647	3.7	$C_{22}H_{36}O_2$	Docosatetraenoic acid	0.017	13.51
327.2334	3.7	$C_{22}H_{32}O_2$	Docosahexaenoicacid	0.017	3.31
337.3117	3.7	$C_{22}H_{42}O_2$	Docosenoic acid	0.017	14.93
671.467	3.6	$C_{37}H_{69}O_8P$	GP34:2	0.017	14.57
498.2904	4.2	$C_{26}H_{45}NO_6S$	Dihydroxcholanoyltaurine	0.018	0.55
145.0507	4.0	$C_6H_{10}O_4$	Adipate	0.020	0.79
133.0506	12.7	$C_5H_{10}O_4$	Deoxyribose	0.021	19.58
317.2491	4.0	$C_{21}H_{34}O_2$	Hydroxypregnone	0.024	7301.99
242.1765	7.1	$C_{13}H_{25}NO_3$	N-Undecanoylglycine	0.026	0.80
467.3537	3.8	$C_{31}H_{48}O_3$	3-Hydroxy-vitamin K	0.028	94.98
371.2595	3.5	$C_{24}H_{36}O_3$	Hydroxycholatrienoic acid	0.028	>10000
480.3105	4.4	$C_{23}H_{48}NO_7P$	C18:0 LPE	0.029	66.51
816.5766	3.9	$C_{44}H_{84}NO_{10}P$	PS38:1	0.029	197.05
303.2335	3.7	$C_{20}H_{32}O2$	Eicosatetraenoic acid	0.030	2.02
521.9842	18.8*	$C_{10}H_{16}N_5O_{14}P_3$	GTP	0.030	11029.57
343.2648	3.9	$C_{23}H_36O_2$	Cholatriene diol	0.032	>10000
442.0178	17.4*	$C_{10}H_{15}N_5O_{11}P_2$	GDP	0.032	>10000
256.0961	14.3*	$C_8H_{20}NO_6P$	GPC	0.032	940.64
145.0145	14.9*	$C_5H_6O_5$	Oxoglutarate	0.033	0.96
861.55	3.6	$C_{45}H_{83}O_{13}P$	PI 36:0	0.037	424.28
661.5195	4.2	$C_{37}H_{75}O_7P$	PA 34:0	0.038	34.04
838.5613	3.6	$C_{46}H_{82}NO_{10}P$	PS 40:4	0.038	97.00
911.5655	3.6	$C_{49}H_{85}O_{13}P$	PI40:5	0.040	626.33
329.249	3.7	$C_{22}H_{34}O_2$	Docosapentaenoic acid	0.040	6.24
810.5298	3.6	$C_{44}H_{78}NO_{10}P$	PS38:4	0.041	61.79
645.4985	4.2	$C_{35}H_{71}N_2O_6P$	SM(d18:1/12:0)	0.044	145.30
159.03	14.0	$C_6H_8O_5$	2-Oxoadipate	0.044	9.37
909.5501	3.6	$C_{49}H_{83}O_{13}P$	PI40:6	0.045	465.81
465.3049	3.6	$C_{27}H_{46}O_4S$	Cholesterol sulfate	0.045	35.00
825.5656	3.5	$C_{46}H_{83}O_{10}P$	PG40:4	0.045	415.16
885.5503	3.6	$C_{47}H_{83}O_{13}P$	PI38:4	0.047	228.57

466.3313	4.5	$C_{23}H_{50}NO_6P$	18:0 LPE ether	0.047	>10000
476.279	7.1	$C_{23}H_{44}NO_7P$	18:2 LPE	0.048	>10000
817.5037	3.5	$C_{46}H_{75}O_{10}P$	GP40:8	0.048	16.22
698.5135	3.9	$C_{39}H_{74}NO_7P$	PE36:2 ether	0.048	79.82
913.5807	3.6	$C_{49}H_{87}O_{13}P$	PI40:4	0.049	411.77
164.0718	10.1*	$C_9H_{11}NO_2$	Phenylalanine	0.050	1.15
712.529	3.9	$C_{40}H_{76}NO_7P$	PC32:2 ether	0.050	162.57
802.5609	3.9	$C_{43}H_{82}NO_{10}P$	GP36:0	0.051	199.53
673.5296	4.1	$C_{37}H_{75}N_2O_6P$	SM 34:2	0.051	113.63
723.4982	3.6	$C_{41}H_{73}O_8P$	GP38:4	0.052	31.06
492.3469	4.3	$C_{25}H_{52}NO_6P$	20:1 LPE	0.052	36.05
464.3153	4.4	$C_{23}H_{48}NO_6P$	18:1 LPE	0.052	77.11
409.2367	4.6	$C_{19}H_{39}O_7P$	LGP16:0	0.053	73.25
436.2841	4.5	$C_{21}H_{44}NO_6P$	LPE 16:1	0.053	>10000
863.5652	3.6	$C_{45}H_{85}O_{13}P$	PI 36:2	0.054	287.24
171.0066	14.4*	$C_3H_9O_6P$	Glycerol 3-phosphate	0.054	96.81
836.5424	3.6	$C_{46}H_{80}NO_{10}P$	PS40:5	0.054	77.51
485.2684	4.4	$C_{25}H_{43}O_7P$	PA(20:4(5Z,8Z,11Z,14Z)e/2:0)	0.054	>10000
786.5299	3.6	$C_{42}H_{78}NO_{10}P$	PS36:2	0.054	110.77
819.5189	3.5	$C_{46}H_{77}O_{10}P$	PG40:7	0.055	>10000
701.5611	4.2	$C_{39}H_{79}N_2O_6P$	hexadecanoylsphingeninephosphocholine	0.055	4.30
821.5349	3.5	$C_{46}H_{79}O_{10}P$	PG40:6	0.055	>10000
726.5452	3.8	$C_{41}H_{78}NO_7P$	PE36:2 ether	0.056	18.58
769.5028	3.5	$C_{42}H_{75}O_{10}P$	PG36:4	0.058	>10000
883.5344	3.6	$C_{47}H_{81}O_{13}P$	PI38:5	0.058	671.92

Table S18 Metabolites in positive ion in medium cardol treated versus control

Row m/z	Row retention time	Name	p-value S1/5	S1/5
813.684	4.1	SM(d18:1/24:1(15Z))	0.000	0.0987
703.5748	4.2	[SP (16:0)] N-(hexadecanoyl)-sphing-4-enine-1-phosphocholine	0.036	5.5843
836.5432	3.6	[PS (18:0/22:6)] 1-octadecanoyl-2-(4Z,7Z,10Z,13Z,16Z,19Z-docosahexaenoyl)-sn-glycero-3-phosphoserine	0.042	#DIV/0!
496.3397	7.1	[PC (16:0)] 1-hexadecanoyl-sn-glycero-3-phosphocholine	0.044	21.1011
812.6157	3.8	PC(16:1(9Z)/22:2(13Z,16Z))	0.047	10.0926
728.5589	3.8	PE(18:1(11Z)/P-18:1(11Z))	0.048	17.4140
742.5745	3.9	[PC (16:1/18:2)] 1-(1Z-hexadecenyl)-2-(9Z,12Z-octadecadienoyl)-sn-glycero-3-phosphocholine	0.049	85.8441
142.0263	14.2	Ethanolamine phosphate	0.050	0.2398
771.6092	3.9	demethylmenaquinone-9	0.050	145.9354
772.6214	3.9	[PC (18:1/18:0)] 1-(1Z-octadecenyl)-2-(9Z-octadecenoyl)-sn-glycero-3-phosphocholine	0.050	117.7986
838.6315	3.8	[PC (18:0/22:4)] 1-octadecanoyl-2-(7Z,10Z,13Z,16Z-docosatetraenoyl)-sn-glycero-3-phosphocholine	0.052	137.7170
836.6162	3.8	[PC (18:0/22:5)] 1-octadecanoyl-2-(4Z,7Z,10Z,13Z,16Z-docosapentaenoyl)-sn-glycero-3-phosphocholine	0.052	25.2539
770.6058	3.9	PC(18:1(11Z)/P-18:1(11Z))	0.052	51.3445
435.1745	4.0	Cys-Leu-Cys-Pro	0.054	0.3983
830.5694	3.8	PC 40:8	0.054	143.2935
834.6003	3.8	[PC (18:1/22:5)] 1-(11Z-octadecenoyl)-2-(7Z,10Z,13Z,16Z,19Z-docosapentaenoyl)-sn-glycero-3-phosphocholine	0.055	20.9977
913.5796	3.6	PI(18:0/22:5(4Z,7Z,10Z,13Z,16Z))	0.055	#DIV/0!
810.6008	3.8	[PC (18:1/20:3)] 1-(9Z-octadecenoyl)-2-(5Z,8Z,11Z-eicosatrienoyl)-sn-glycero-3-phosphocholine	0.055	15.1465
832.5847	3.8	PC40:7	0.055	78.2575
840.5749	3.5	1-22:1-2-18:3-phosphatidylserine	0.055	#DIV/0!
786.6008	3.9	[PC (18:1/18:1)] 1-(9Z-octadecenoyl)-2-(9Z-octadecenoyl)-sn-glycero-3-phosphocholine	0.056	12.7335
